# Recent Progress on Flexible Room-Temperature Gas Sensors Based on Metal Oxide Semiconductor

**DOI:** 10.1007/s40820-022-00956-9

**Published:** 2022-10-21

**Authors:** Lang-Xi Ou, Meng-Yang Liu, Li-Yuan Zhu, David Wei Zhang, Hong-Liang Lu

**Affiliations:** 1 State Key Laboratory of ASIC and System, Shanghai Institute of Intelligent Electronics &Systems, School of Microelectronics, Fudan University, Shanghai, 200433 People’s Republic of China; 2 Yiwu Research Institute of Fudan University, Chengbei Road, Yiwu City, 322000 Zhejiang People’s Republic of China

**Keywords:** Metal oxide semiconductor, Flexible gas sensor, Room temperature, Nanomaterials

## Abstract

With the rapid development of the Internet of Things, there is a great demand for portable gas sensors. Metal oxide semiconductors (MOS) are one of the most traditional and well-studied gas sensing materials and have been widely used to prepare various commercial gas sensors. However, it is limited by high operating temperature. The current research works are directed towards fabricating high-performance flexible room-temperature (FRT) gas sensors, which are effective in simplifying the structure of MOS-based sensors, reducing power consumption, and expanding the application of portable devices. This article presents the recent research progress of MOS-based FRT gas sensors in terms of sensing mechanism, performance, flexibility characteristics, and applications. This review comprehensively summarizes and discusses five types of MOS-based FRT gas sensors, including pristine MOS, noble metal nanoparticles modified MOS, organic polymers modified MOS, carbon-based materials (carbon nanotubes and graphene derivatives) modified MOS, and two-dimensional transition metal dichalcogenides materials modified MOS. The effect of light-illuminated to improve gas sensing performance is further discussed. Furthermore, the applications and future perspectives of FRT gas sensors are also discussed.

## Highlights

- Latest progress on flexible room temperature (FRT) gas sensor based on metal oxide semiconductors (MOS) is comprehensively reviewed.
- FRT gas sensor based on pristine MOS and MOS modified with noble metal nanoparticles, organic polymers, carbon based materials and transition metal dichalcogenide materials are meticulously reviewed.
- The gas sensing mechanism of MOS chemiresistive gas sensors are introduced and the applications, future perspectives, and challenges of FRT gas sensors are also proposed.

## Graphical abstract

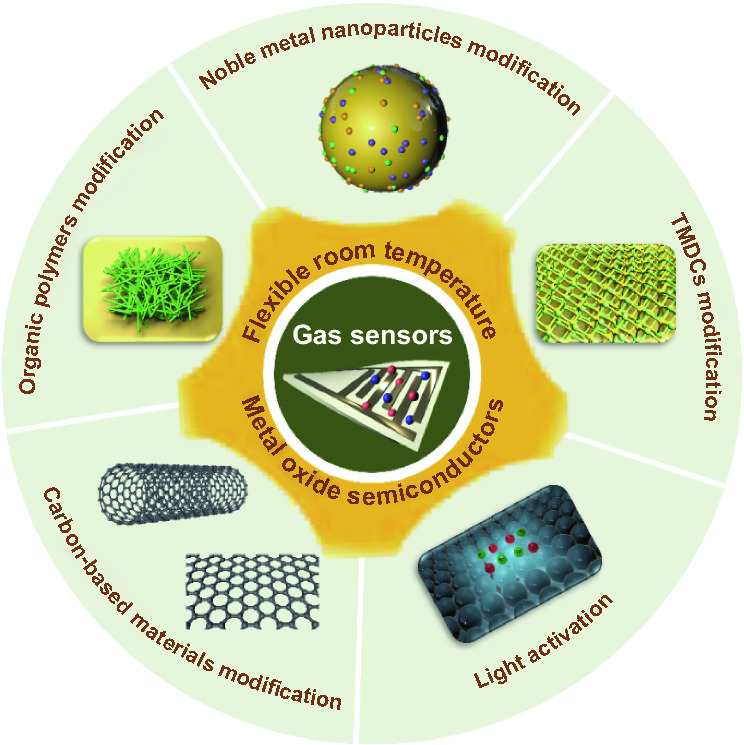

## Introduction

In the past decade, the Internet of Things (IoTs), the networks that connects diverse sensors and actuators, has attracted enormous attention [1–5]. Traditional electronic sensors are gradually transforming from bulky solid-state devices to portable, high-performance, and multifunctional devices. The rapid development of flexible electronics makes them play a significant role in the wide applications of IoTs, which serves as an ideal platform for wearable devices [6–12]. In recent years, flexible and wearable devices have been used as attractive alternatives to bulky analytical instruments and applied to perform continuous physiological monitoring of body movement, blood temperature, blood glucose, heart rate, and electrophysiological activities such as electroencephalography, electrocardiography, and electromyography [13–16]. Recently, metaverse has become a hot issue. Metaverses are sensory-rich virtual worlds where people engage with each other as virtuous avatars without any physical limitations [17, 18]. Wearable devices integrating kinds of sensors, which can conduct continuous physiological monitoring and real-time interaction with software agents, are of vital importance to the rapid development of metaverse [19].

Various hazardous gases are released from industrial and agricultural processes, such as CO, NO_x_, NH_3_, H_2_, H_2_S, and volatile organic compounds (VOCs), including ethanol, isopropanol, acetaldehyde, and formaldehyde [20–23]. The leakage of these pollutant gases will not only pollute the environment, but also have a detrimental effect on human body [24–27]. For instance, emissions of NO_x_ from coal fired power stations lead to ozone holes, acid rain and severe haze in metropolitan areas, causing serious damage to human health, the ecological environment and the national economy [28–30]. In particular, serious air pollution can damage the lungs of humans, facilitating the transmission and infection of COVID-19 [31–34]. Real-time detection of toxic gases in industrial production and the development of wearable gas warning devices are of significant to workers, especially in environments where toxic gas leaks can occur. In addition to the need for timely detection of hazardous gases, the detection of specific gases is also widely used in the area of medical healthcare [35]. Gas chromatography–mass spectrometry analysis of human exhaled gas showed that the exhaled gas contains more than 870 different VOCs [36–38]. It is noticeable that the presence of some specific VOCs is related to specific diseases [39–41]. Through a simple breath analysis, many diseases can be diagnosed and therapeutic monitored noninvasively [42, 43]. For instance, ammonia and fatty acids are found in the breath of patients with cirrhosis, while acetone and isoprene are found in the breath of patients with diabetes [44–46]. However, the conventional technology of breath analysis requires bulky and expensive equipment, long time-consumption and well-trained personnel. Therefore, there is an increasing requirement for high-performance gas sensors with low-cost, high sensitivity, rapid response, fabulous selectivity, and low limit of detection (LOD).

Mechanically flexible gas sensors are one of the most popular and forefront research directions of IoTs, meeting the enormous industrial requirements of smart wearable devices [47]. Moreover, they are crucial for monitoring environmental gases, gaseous pollutants, volatile hazards, humidity, exhaled gases, body odor, nerve agents or explosives, and food quality. Conventional gas sensors are typically manufactured on inorganic substrates, including quartz, glass, alumina ceramic tubes and silicon wafers. However, their rigidity and fragility limit their application in a variety of new fields. In contrast, the integration of gas sensors on flexible substrates, such as polymer, textiles, and paper-based substrates has attracted the increasing attention of researchers over the past few years, making them highly promising in the fields of portable electronics [48–51], smart textiles [52–54], radio frequency identification (RFID) [55–59], and medical health [60–62]. However, the sensing performance of flexible gas sensors including response value, selectivity, response/recovery time, and LOD is largely influenced by operating temperature, which generally require the configuration of microheaters, resulting in high energy consumption, great complexity of microstructure, and limitations of applications [63]. Therefore, the flexible gas sensors operating at room temperature (RT) are gradually arousing extensive attention. Their portability, excellent mechanical flexibility in harsh environments, and low energy consumption make them promising for various applications. Recently, flexible room-temperature (FRT) MOS-based sensors have been reported to detect a variety of gases, including NO_2_ [54, 64–78], NH_3_ [79–89], H_2_ [90–94], H_2_S [68, 95–98], C_2_H_2_ [99], ethanol [100–103], acetaldehyde [104], formaldehyde [105], acetone [106], ozone [107], isopropanol [108], trimethylamine [109], and triethylamine [110, 111]. Beyond that, some reported FRT gas sensors have been applied to practical applications, such as smart face masks [100], E-textiles [54, 104], passive wireless RFID [112], disease detection [98], and large-scale flexible sensors array [65, 113], exhibiting broader application prospects in the fields of IoTs, metaverses, industrial production, medical application, etc. Nowadays, personalized wearable FRT gas sensors are extensively employed to monitor the exhaled gas of patients while they are out of hospital and provide sufficient information [48, 114]. For example, ketogenic diets (KDs) can perform more effective weight loss when cooperated with a FRT acetone gas sensor monitoring the concentration of exhaled acetone [115, 116].

**Fig. 1:**
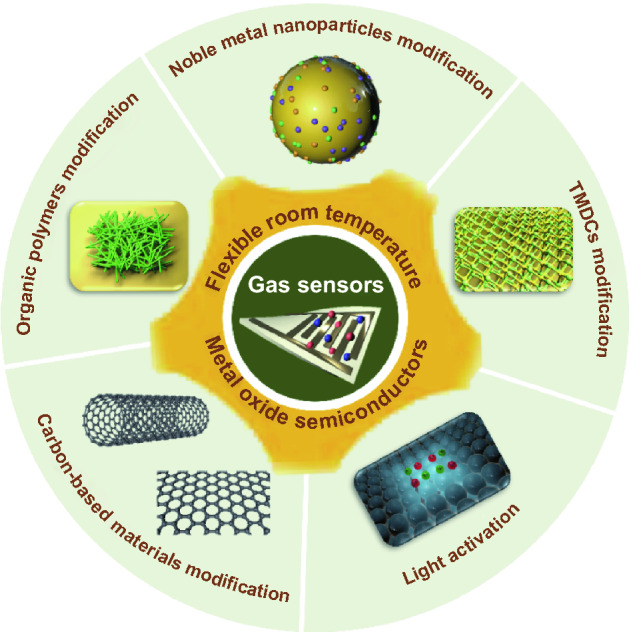
The strategies to achieve high-performance FRT MOS-based chemiresistive gas sensors

The FRT gas sensors have high requirements for sensing materials that not only perform well at RT, but also under bending conditions. In general, traditional gas sensing materials including metal oxide semiconductor (MOS), conducting polymers, and carbon-based materials [117–122]. Among them, MOS is the most popular commercial sensing material due to its merits of easy synthesis, high response value, low cost, short response/recovery time, great reversibility, and excellent stability [123–129]. However, its disadvantages such as high-temperature operation and high-power consumption hinder its wearable applications [130–132]. What’s worse, high-temperature operation not only degrades the nanostructure of the sensing material, deteriorating the gas sensing performance, but also hinders the detection of explosive or flammable gases. Therefore, the ability of the MOS-based sensors to work at RT is of vital significance because it leads to very low power consumption and simplifies the sensor structure. At the meantime, the conducting polymers-based sensors can operate at RT without additional power requirements, but their performance degrades in humid environment. In addition, the carbon-based materials can also greatly lower the operating temperature and contribute to high sensitivity, but long response/recovery time and complex processes render them inadequate for wide application [98, 133–135]. To achieve high-performance FRT MOS-based chemiresistive gas sensors, optimization has been performed from material design and alternate activation, which is graphically presented in Fig. 1. The thought of combining MOS with conducting polymers or carbon-based materials is proposed owing to the integrated advantages of both components [136–142]. Beyond that, a number of strategies have been used to improve sensing performance of MOS-based FRT gas sensors up to now, such as morphological modification of pristine MOS, noble metal nanoparticles modified MOS, light-illuminated MOS, and two-dimensional (2D) transition metal dichalcogenides (TMDCs) modified MOS. Table 1 summarizes various reported MOS-based FRT sensors.

**Table 1:** Sensing performance of various MOS-based FRT gas sensors

Material	Structure	Substrate	Synthesis method	Target gas	C (ppm)	Response	τ_res_/τ_rec_	LOD	Bending cycles	Response decrease/bending cycles	References
*Part A: Sensing performance of pristine MOS FRT gas sensors*											
ZnO	Nanowires	PET	Hydrothermal	H_2_	1,000	5^c^	~ 600/s	–	–	–	[90]
ZnO	Nanorods	Nylon	Hydrothermal	H_2_	500	109^c^	149/122 s	–	–	–	[92]
ZnO	Nanoparticles	PET	–	Ethanol	800	2.2^a^	–	–	–	–	[102]
ZnO_1-x_	Sheet-like	PP	Suspension flame spraying	NO_2_	1	2.568^b^	60/230 min	0.25 ppm	–	–	[70]
ZnO	Nanoparticles	Cotton fabrics	Sol–gel	Acetaldehyde	100	33^c^	228/14 s	–	–	–	[104]
In_2_O_3_	Cubic crystals	PVA	Hydrothermal	Ethanol	100	~ 1.4^a^	5/3 s	10 ppm	–	–	[103]
In_2_O_3_	Octahedral nanopowders	PI	Oxidation	NO_2_	5	5.75^a^	105/785 s	3 ppm	–	–	[69]
In_2_O_3_	Nanowires	PI	Electrospun	NO_2_	0.5	5.52	–	10 ppb	–	–	[78]
InO_x_	Thin films	PET	Magnetron sputtering	Ozone	1.07	~ 72.5^c^	7/min	15 ppb	–	–	[107]
TiO_2_	Thin films	PET	Spinning	Formaldehyde	5	> 570^b^	9/ ~ 300 s	3.8 ppb	200	No obvious decrease	[105]
TiO_2_	Nanotubes	PI	Anodization	TMA	400	150^c^	~ 25/s	40 ppm	–	–	[109]
CuO	Nanorectangles	PET	Hydrothermal	NH_3_	5	~ 0.25^b^	90/120 s	5 ppm	–	–	[82]
Cellulose/Fe_2_O_3_	Nanoparticles	PET	Hydrothermal	NO_2_	200	~ 1,100^c^	50/30 s	2 ppm	–	–	[71]
WO_3-δ_	Films	PI	Granule spray	NO_2_	10	18,500^c^	17/25 s	1.88 ppm	4,000	< 66.7%/4,000	[76]
SnO_2_/ZnO	Nanofibers	PET/PDMS/Paper	Electrospun	NO_2_	0.1	56^c^	–	0.1 ppm	–	–	[233]
*Part B: Sensing performance of noble metal nanoparticles modified MOS FRT gas sensors*											
Ga/ZnO	Nanorods	PI	Hydrothermal	H_2_	1,000	91^c^	~ 18.8/s	0.2 ppm	–	–	[91]
Pd/ZnO	Nanorods	PI/PET	Hydrothermal	H_2_	1,000	91.2^c^	~ 18.8/s	0.2 ppm	10^6^	13%/10^5^, 52%/10^6^	[93]
Ag/ZnO	Nanorods	PI	Hydrothermal	C_2_H_2_	1,000	26.2^a^	66/68 s	3 ppm	10^4^	10.6%/10^4^	[99]
Pt/SrGe_4_O_9_	Nanotubes	PET	Electrospun	NH_3_	100	7.08^a^	17/16 s	1 ppm	1,000	No obvious decrease	[80]
PANI/Rh/SnO_2_	Nanotubes	PET	Electrospun	NH_3_	100	13.6	113/159 s	500 ppb	1,000	21%	[172]
PANI/In_2_O_3_/Au	Nanospheres/Nanofibers	PI	Hydrothermal	NH_3_	100	46^a^	118/144 s	–	100	No obvious decrease	[79]
SWCNT/PdO/Co_3_O_4_	Nanocubes	PI	Chemical precipitation	NO_2_	20	27.33^c^	–	1 ppm	4,000	No obvious decrease	[74]
*Part C: Sensing performance of organic polymers modified MOS FRT gas sensors*											
PANI/WO_3_	Nanofibers/Flowerlike	PET	In situ polymerization	NH_3_	10	7^a^	13/49 s	500 ppb	–	–	[87]
PANI/CeO_2_	Nanosheet/Nanoparticle	PI	Self-assembly	NH_3_	50	262.7^c^	14/6 min	16 ppb	500	No obvious decrease	[83]
PANI/α-Fe_2_O_3_	Nanofiber/Nanoparticle	PET	Sol–gel	NH_3_	100	72^c^	50/1575 s	2.5 ppm	–	–	[81]
PANI/Fe_2_O_3_	Sea cucumber-shaped	PET	Hydrothermal	NH_3_	100	6.12^a^	100/s	0.5 ppm	–	–	[85]
PANI/α-Fe_2_O_3_	Nanofiber/Nanoparticle	PET	In situ polymerization	NH_3_	100	39^c^	27/46 s	5 ppm	–	–	[89]
PANI/CoFe_2_O_4_	Nanofiber/Nanoparticle	PET	In situ polymerization	NH_3_	50	118.3^c^	24.3/ ~ 410 s	25 ppb	500	3.4%/500	[84]
SnO_2_@PANI	Nanoparticles/Nanofiber	PET	Hydrothermal	NH_3_	100	29.8	125/167 s	10 ppb	100	No obvious decrease	[234]
PANI@SnO_2_	Nanoparticle/Nanofibers	PET	In situ polymerization	NH_3_	100	29^a^	34/s	> 1.8 ppm	–	–	[86]
PANI/SnO_2_	Nanofibrous/Nanoparticle	PET	In situ polymerization	TEA	100	69^a^	~ 5/s	1.2 ppm	–	–	[110]
PANI/MoO_3_	Nanorods/Nanoparticle	PET	In situ polymerization	TEA	100	22.6^a^	35/88.42 s	0.55 ppm	–	–	[111]
PANI@SnO_2_/Zn_2_SnO_4_	Nanofiber/Nanosphere	PET	In situ polymerization	NH_3_	100	20.4	46/54 s	500 ppb	500	26.5%/500	[186]
ZnO/S, N: GQDs/PANI	Nanopolyhedra/Nanorob	PET	In situ polymerization	Acetone	0.5	2^c^	15/27 s	0.1 ppm	60	No obvious decrease	[106]
Chitosan/WO_3_/IL	Nanocomposite membranes	Chitosan/IL	Sol–gel	H_2_S	200	2.75^a^	~ 13.6/s	15 ppm	–	–	[97]
CMC/CuO/IL	Nanocomposite membranes	CMC/IL	Hydrothermal	H_2_S	300	~ 20^c^	~ 52	15 ppm	–	–	[95]
PVA/WO_3_/IL	Nanocomposite membranes	PVA/IL	Sol–gel	H_2_S	300	~ 12^c^	~ 19.1/s	10 ppm	5	No obvious decrease	[96]
*Part D: Sensing performance of carbon-based materials modified MOS FRT gas sensors*											
MWCNT/WO_3_	Nanoparticles	PET	Hydrothermal	NO_2_	5	14^c^	10/27 min	0.1 ppm	10^8^	0.7%/10^6^, 2.1%/10^8^	[73]
MWCNT/WO_3_/RGO	Nanoparticles	PI/PET	Hydrothermal	NO_2_	5	17^c^	7/15 min	1 ppm	10^6^	No obvious decrease	[72]
MWCNT/Co_3_O_4_	Nanofiber	Textile fabric	Heat treatment	NO_2_	1,000	~ 32^c^	–	0.1 ppm	–	–	[64]
SWCNT/Fe_2_O_3_	Nanospheres	PP	CVD	NO_2_/H_2_S	10/100	19/18.3^c^	300/300 s	1 ppm	16	No obvious decrease	[68]
SWCNT/CuO	Flower-shaped	PP	Hydrothermal	H_2_S	1	~ 35^c^	7/28 s	100 ppb	–	–	[112]
SWCNT@ZnO	Quantum dot	Nylon	Redox reaction	Ethanol	500	1.09^a^	992/301 s	–	1,000	No obvious decrease	[100]
RGO/WO_3_·0.33H_2_O	Nanoneedles	PET	Hydrothermal	Isopropanol	100	4.96^a^	60/s	1 ppm	100	No obvious decrease	[108]
In_2_O_3_@RGO	Nanoparticles	PI	–	NO_2_	1	31.6^c^	4.2/13.3 min	50 ppb	–	–	[65]
RGO/ZnO	Nanosheet	Cotton/elastic threads	–	NO_2_	15	44^c^	140/630 s	0.2 ppm	3,000	No obvious decrease	[54]
Graphene/CdO	Nanoparticles	–	–	LPG	600	77^c^	–	100 ppm	–	–	[213]
RGO/SnO_2_	Nanoparticles	PI	Spraying	NO_2_	100	0.2640^b^	412/587 s	20 ppm	1,000	10.3%/1,000	[231]
RGO/SnO_2_/PVDF	Hot-press thick film	PVDF	Hot press	H_2_	100	49.2^c^	34/142 s	0.5 ppm	–	–	[94]
SnO_2_/RGO/PANI	Hollow spheres	PET	In situ polymerization	H_2_S	2	60.11^c^	82/78 s	50 ppb	60	No obvious decrease	[98]
*Part E: Sensing performance of TMDCs materials modified MOS FRT gas sensors*											
SnO_2_/SnS_2_	Nanotubes	PET	Hydrothermal	NH_3_	100	2.48^a^	21/110 s	1 ppm	3,000	No obvious decrease	[88]
SnO_2_/MoS_2_	Thin films	PET	E-beam evaporation	NO_2_	9	7.57	–	3 ppm	–	–	[222]
Au/SnO_2_/WS_2_	Nanotubes	PI	Sol–gel	NH_3_	50	3.687	~ 180/ ~ 330 s	0.5 ppm	10,000	20.26%/1,0000	[221]
*Part F: Sensing performance of inorganic materials modified MOS FRT gas sensors*											
In_2_O_3_/g-C_3_N_4_	Nanofibers	Yttria-stabilized zirconia	ALD	NO_2_	1	7.2^a^	31/44 s	50 ppb	–	–	[66]
*Part G: Sensing performance of IGZO based thin-film transistors FRT gas sensors*											
IGZO	Thin films	PI	CVD	NO_2_	5	~ 1.3^a^	–	2 ppm	–	–	[75]
IGZO	Nanofiber network	PEDOT:PSS	Blow-spinning	NO_2_	20	33.2^c^	5/5 s	20 ppb	1,000	No obvious decrease	[67]

C: concentration; τ_res_/τ_rec_: response time/recovery time; Response^a^ = *R*_a_/*R*_g_ (reducing gas) or *R*_g_/*R*_a_ (oxidizing gas); Response^b^: Δ*R*/*R*_g_ (reducing gas) or Δ*R*/*R*_a_ (oxidizing gas); Response^c^: Δ*R*/*R*_g_** × **100% (reducing gas) or Δ*R*/*R*_a_** × **100% (oxidizing gas). *R*_a_: resistance of the sensors exposed to the background gas, *R*_g_: resistance of the sensors exposed to the target gas, Δ*R*: the change in resistance of the sensors after exposure to the target gas

*PET* Poly(ethylene terephthalate); *PP* Polypropylene; *PVA* Polyvinyl alcohol; *PI* Polyimide; *PDMS* Poly(dimethyl siloxane); *PVDF* Polyvinylidene fluoride; *PEDOT:PSS* Poly(3,4-ethylenedioxythiophene) polystyrene sulfonate; *IL* Ionic liquid; *CMC* Carboxymethyl cellulose; *TMA* Trimethylamine; *TEA* Triethylamine; *LPG* Liquid petroleum gas; *PANI* Polyaniline; *GQD* Graphene quantum dots; *SWCNT* Single-walled carbon nanotubes; *MWCNT* Multi-walled carbon nanotubes; *Mxene* Transition metal carbides and carbonitrides; *RGO* Reduced graphene oxide; *IGZO* Indium gallium zinc oxide

Several reviews based on flexible gas sensors have also been published, introducing carbon-based [49, 143, 144], organic polymers-based [145], and TMDC-based [146] gas sensors. In addition, several reviews dedicated to RT gas sensors have been published, discussing the development of various nanostructured materials-based RT gas sensors [120, 132, 147, 148] and MOS-based chemiresistive RT gas sensors [118, 149]. However, no comprehensive review focusing on the recent advances on MOS-based FRT gas sensors is available. Consequently, this review will systematically summarize and analyze the sensing mechanisms and recent advances in FRT MOS-based gas sensors based on pristine MOS, noble metal nanoparticles modified MOS, organic polymers modified MOS, carbon-based materials modified MOS, TMDCs materials modified MOS. In addition, the effect of light-illuminated on improving the gas sensing performance is further discussed. The current applications of FRT gas sensors are also summarized.

## Gas Sensing Mechanism of MOS Chemiresistive Gas Sensors

### Pristine MOS Gas Sensing Mechanism

The gas sensing mechanism of MOS is based on the oxygen adsorption model, which assumes that the change in resistance is related to chemisorbed oxygen [150–152]. In air, oxygen molecules adsorb on the MOS surface and form negatively charged chemisorbed oxygen (${\text{O}}_{2}^{-}$, O^−^, O^2−^) by trapping conduction band electrons. The type of chemisorbed oxygen is related to the operating temperature and species of MOS material, which significantly determines the sensing performance of the sensing material [153, 154]. However, the temperature interval corresponding to the presence of chemisorbed oxygen ions on metal oxides is not well known and varies from different metal oxides. In general, ${\text{O}}_{2}^{-}$ is usually chemisorbed when the temperature is below 100 °C. Once the temperature is between 100 and 300 °C, O^−^ is generally chemisorbed and ${\text{O}}_{2}^{-}$ disappears rapidly. And when the temperature exceeds 300 °C, the chemisorbed oxygen is mainly in the form of O^2−^ [155]. The process of oxygen ion formation can be summarized by the following equations [156]:

1 $${\text{In}}\;{\text{air}}:\;{\text{O}}_{2 } ({\text{gas}}) \to {\text{O}}_{2 } \left( {{\text{ads}}} \right)$$

2 $${\text{T}} < 100 \,^\circ {\text{C}}: {\text{O}}_{2} ({\text{ads}}) + e^{ - } \to {\text{O}}_{2}^{ - } ({\text{ads}})$$

3 $$100 \;^\circ {\text{C}} < {\text{T}} < 300\;^\circ {\text{C}}:{\text{O}}_{2}^{ - } ({\text{ads}}) + e^{ - } \to 2{\text{O}}^{ - } ({\text{ads}})$$

4 $${\text{T}} > 300 \,^\circ {\text{C}}:{\text{O}}^{ - } ({\text{ads}}) + e^{ - } \to {\text{O}}^{2 - } ({\text{ads}})$$

As a result of oxygen adsorption, an electron depletion layer with a low electron concentration is formed on the surface of the n-type MOS, which has a higher resistance than the core region due to the reduced number of electrons. While on the surface of the p-type MOS, a hole accumulation layer is formed, which has a lower resistance than the core region of the MOS due to the increased number of holes.

When exposed to the target reducing gases, the target molecules adsorb to the surface of MOS sensing layer and react with the chemisorbed oxygen ions, releasing electrons into the MOS material. Conversely, for oxidizing gases, more electrons are trapped from the MOS surface. As a result of these processes, the resistance of the sensing layer will change significantly, which will result in the response of the gas sensor. Therefore, the sensitivity of the MOS-based gas sensors is generally defined as *R*_a_/*R*_g_ (reducing gas), *R*_g_/*R*_a_ (oxidizing gas), ΔR/R_g_ (reducing gas), Δ*R*/*R*_a_ (oxidizing gas), Δ*R*/*R*_g_ × 100% (reducing gas), or Δ*R*/*R*_a_ × 100% (oxidizing gas) (where *R*_a_ is the resistance of the sensors exposed to the background gas, *R*_g_ is the resistance of the sensors exposed to the target gas, Δ*R* is the change in resistance of the sensors after exposure to the target gas).

Conventionally, MOS-based gas sensors are operated at 300–500 ℃ to provide sufficient activation energy to facilitate oxygen adsorption, which is also called thermal activation. In contrast, at RT, the chemisorbed oxygen ions on the MOS surface are mainly ${\text{O}}_{2}^{-}$, with a low content of other chemisorbed oxygen ions, making it a challenge to achieve RT operation of MOS. From the perspective of material design, in order to achieve RT operation, pristine MOS materials can be nanoconstructed with different morphologies, facilitating efficient modulation of the electron depletion layer. In addition, the construction of heterogeneous structures by surface modification of MOS materials is also an efficient strategy.

### Heterostructured MOS-Based Nanocomposites Gas Sensing Mechanism

Generally, MOS are often hybrids with other MOS, organic polymers, carbon-based materials and TDMCs to significantly improve their sensing performance. Due to the different energy band structures of MOS and hybrid materials, electrons or holes are transferred at the interface between the components until their Fermi levels equilibrate to the same energy level. This process results in the formation of heterojunctions at the interface between the MOS and the hybrid materials. The formation of the heterojunctions modulates the thickness of the depletion/accumulation layer and the height of the potential barrier, changing the internal electron distribution between different components and significantly affecting the sensing performance of the sensing materials. When analyzing the mechanism of MOS nanocomposites, the effect of heterojunctions needs to be considered primarily. In this section, the mechanism of heterostructured MOS-based nanocomposites is illustrated with two subsections: anisotype heterojunction (p–n) and isotype heterojunctions (n–n, p–p).

For the anisotype heterojunction (p–n), the Fermi energy level of the n-type semiconductor is generally higher than the Fermi energy level of the p-type semiconductor. Therefore, when two dissimilar materials with different Fermi levels contact, the electrons are transferred from the n-type semiconductors to the p-type semiconductors, and the holes are transferred from the p-type semiconductors to the n-type semiconductors until the Fermi energy levels are balanced. After that, a depletion layer is formed at their interface and the energy bands on both sides are bent to create a potential barrier, which makes the electron transport channel narrower. In addition, owing to the wider depletion layer width of the heterojunction, the initial resistance increases greatly compared to the pristine MOS. Therefore, when exposed to an oxidizing gas atmosphere, the increase in resistance is small. However, when exposed to a reducing gas atmosphere, the resistance decreases sharply, which can improve the selectivity of the sensor to some extent.

For the isotype heterojunction (n–n, p–p), the band bending phenomenon also occurs due to the difference in Fermi energy levels. For n–n heterojunctions, electrons are transferred from the side with high Fermi energy levels to the side with low Fermi energy levels, while an electron depletion layer is formed on the side with high Fermi energy levels and an electron accumulation layer is formed on the other side. Similarly, for p–p heterojunction, holes are transferred from the side with low Fermi energy levels to the side with high Fermi energy levels, while the hole accumulation layer is formed on the side with high Fermi energy levels and the hole depletion layer is formed on the other side.

Generally, to achieve RT operation and enhanced sensing performance of MOS-based gas sensors, two perspectives can be considered: material design and alternate activation. From the perspective of material design, for pristine MOS, reducing the grain size, constructing various morphologies with enhanced surface-to-volume ratio are considerable sensitization strategies. In addition, chemical and electronic sensitization can be achieved by decorating precious metals, which can significantly improve the sensing performance under RT. Hybridizing with some unique materials that can chemically react with the target gas is also an effective way to achieve RT operation. For instance, PANI reduces from the conductive emeraldine salt state to the non-conductive intrinsic emeraldine base state when reacting with certain target gases, which improves the sensing performance at RT. The addition of carbon-based materials to the MOS can also remarkably improve the conductivity of the MOS-based nanocomposite and optimize its sensing properties at RT. The modification of 2D TMDCs can effectively modulate the heterojunction between MOS and TMDCs due to its unique surface effect, exhibiting great potential at RT as well. From the perspective of alternate activation, materials with self-heating capability can achieve thermal activation without microheaters. Photoactivation also facilitates the reaction with the target gas by introducing additional photogenerated electron–hole pairs, thus enhancing the gas sensing performance at RT. These specific sensing mechanisms will be discussed further in different chapters.

## Pristine MOS FRT Gas Sensors

**Fig. 2:**
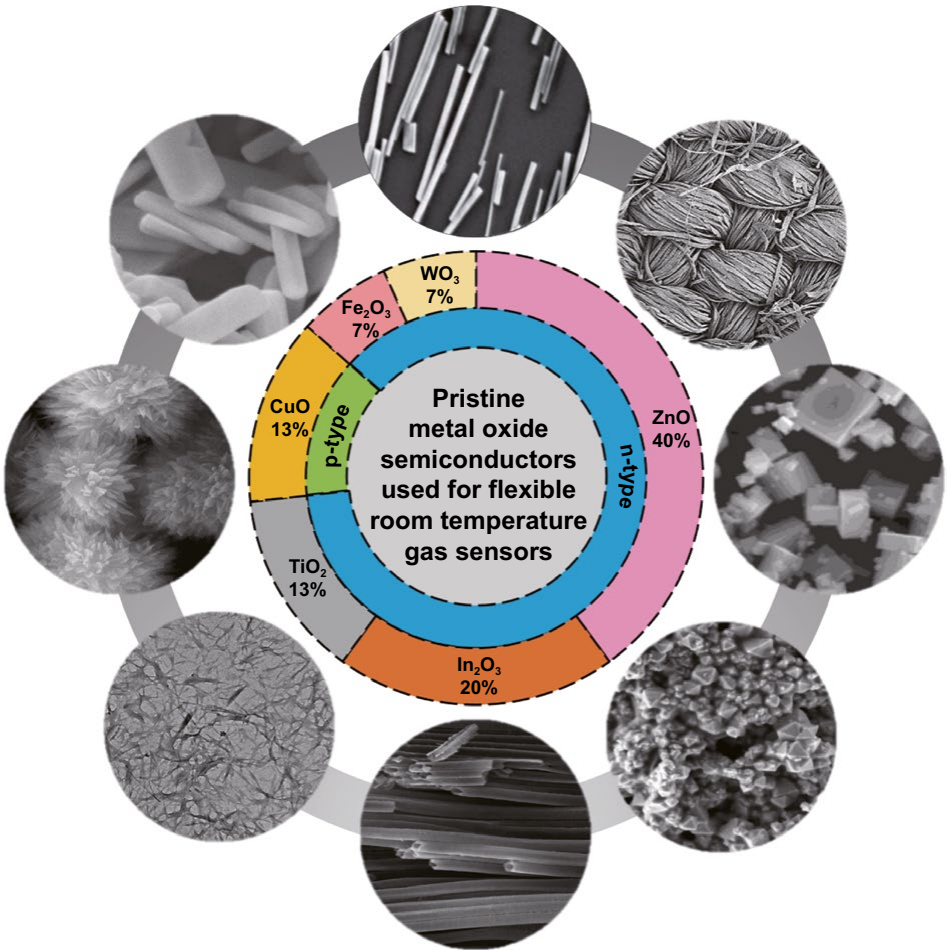
Schematic diagram of various pristine metal oxide semiconductor used for flexible room-temperature gas sensors introduced in this chapter

Pristine MOS generally have great stability, reversibility, and their manufacturing process is facile and cost-effective [157–159]. Various morphologies of nanostructures for pristine MOS including nanoparticles [71, 97, 101, 102, 104], nanorods [92], nanowires [90], nanotubes [109], nanocubic crystals [103], nanorectangles [82], sheet-like [70], and columnar [160] nanostructures have been fabricated and employed in flexible gas sensors to help reducing operating temperature to RT (Fig. 2). These sensors can be used to detect a variety of gases, including NH_3_, NO_2_, H_2_, H_2_S, ethanol, isopropanol, trimethylamine (TMA), formaldehyde, acetaldehyde and ozone, performing with good sensing performance.

N-type pristine MOS including ZnO, In_2_O_3_, WO_3_, Fe_2_O_3_, and TiO_2_ are the sensing materials that have been reported for FRT chemiresistive gas sensors. Among these materials, ZnO has been a fascinating FRT gas sensing material attributed to the fusion of its inherent characteristics such as high electron mobility, wide bandgap, excellent chemical stability, non-toxicity and biocompatibility as well as its versatility in fabricating various nanostructures such as nanorods [92], nanowires [90], nanoparticles [102, 104], columnar [160], and sheet-like [70] nanostructures. Mohammad et al. [92] fabricated hexagonal-shaped and well-aligned ZnO nanorods assembled on nylon substrates using hydrothermal method. The high specific surface area and high crystalline quality of ZnO nanorods contributed to the good response of 109% to 500 ppm hydrogen ambient with rapid response/recovery time. Ong et al. [90] synthesized ZnO nanowires with a simple low-temperature hydrothermal method and proposed three methods, slide transfer, roll transfer and thermal transfer, to transfer the samples onto flexible poly(ethylene terephthalate) (PET) substrates. The mechanically flexible ZnO nanowires gas sensor exhibited an n-type response value of 5.0% to 1000 ppm hydrogen. However, the ZnO nanowires with surface modification of ammonia plasma exhibited p-type hall results, indicating that ammonia plasma treatment can lead to effective conductivity modulation of the ZnO nanowires. Furthermore, the ammonia plasma-treated ZnO nanowires showed a significantly enhanced response of 15% to 500 ppm hydrogen with no apparent degradation after 14 months long-term test. Cotton fabrics have also been reported to be excellent flexible substrates for MOS-based FRT gas sensors. Subbiah et al. [104] reported a multifunctional acetaldehyde gas sensor developed by growing hexagonal-shaped ZnO nanoparticles on cotton fabrics through seed layer enhanced sol–gel techniques. The introduction of cotton fabric contributes to the uniform distribution of ZnO nanoparticles and the high porosity between yarns. More importantly, this ZnO nanostructure-modified cotton fabric is also equipped with ultraviolet (UV) radiation protection, which reveals its promising application in wearable gas sensing devices with UV filtering capability.

In_2_O_3_ is also an outstanding material for FRT gas sensors due to its excellent low-temperature gas sensing properties and the ability to synthesize different controllable morphologies. In_2_O_3_ cubic crystals were prepared by a modified hydrothermal synthesis [103] and then made into a flexible composite film by blending with polyvinyl alcohol (PVA). This flexible composite film exhibited a response of 1.4 to 100 ppm ethanol at RT with significantly rapid response/recovery time of 5/3 s. Nanostructure of In_2_O_3_ octahedral nanopowders [69] was fabricated by oxidation of ionic precursor compound in low-oxygen atmosphere. The nanopowders were further mixed with propanediol and then deposited on polyimide (PI) substrates. The obtained sensor exhibited a good response of 3 to 5 ppm NO_2_ at 100 °C, performing its great potential for FRT NO_2_ sensors. Kiriakidis et al. [107] reported an ozone sensor based on InO_x_ thin films with cylindrical structure and nanosize grains of about 20 nm, grown by magnetron sputtering on PET substrate. The sensor has a fabulous LOD of 15 ppb at RT and exhibited a large response of nearly 72.5% to 15 ppb ozone.

N-type WO_3_, TiO_2_, and Fe_2_O_3_ were also reported for FRT gas sensors, while other n-type pristine MOS applied for FRT gas sensors are rarely reported. Ryu et al. [76] prepared porous WO_3-δ_ films on PI substrate with granule spray process. The gas sensor exhibited high sensing properties with a great response of 18,500% to 10 ppm NO_2_, response/recovery time of 17/25 s and LOD of 1.88 ppm. Furthermore, the sensor maintained high performance after 4,000 bending/relaxing cycles, showing excellent flexibility properties. TiO_2_ has also been reported to be used in the preparation of TMA sensors. TMA is known to be released in dead fishes with a pungent and ammonia-like odor. The detection of ppm-levels TMA has been used to estimate the freshness of fishes and seafood products. However, most of the reported MOS-based TMA sensors work at high temperature, which is seriously inconsistent with the actual application. Perillo et al. [109] reported a TiO_2_ nanotube prepared by anodization method and then slid it onto the PI substrate to obtain a flexible sensor. The response of TiO_2_ FRT sensor reached 150% to 400 ppm TMA with fast response time of 25 s and the detection limit was 40 ppm, meeting the actual application situation. Kim et al. [71] demonstrated a novel bio-friendly renewable NO_2_ sensor based on cellulose nanocrystals (CNC)/Fe_2_O_3_ composites, which exhibited whisker-shaped morphology. The CNC/Fe_2_O_3_ sensor exhibited a tremendous response of nearly 1100% to 200 ppm NO_2_ with fabulous reversibility, which can be contributed to the novel dispersive morphology.

There are few p-type pristine MOS that have been reported in FRT gas sensors due to their low gas response. Hübner et al. [161] demonstrated that the sensitivity of the n-type MOS gas sensor is the square of a p-type gas sensor with identical morphology, suggesting that it is a great challenge to design a pristine p-type MOS gas sensor with tremendous sensing performance. Kuritka et al. [101] fabricated a fully inkjet-printed CuO-based humidity and ethanol sensor on PET substrate. The CuO nanoparticles were fabricated by microwave-assisted solvothermal method and exhibited flowerlike morphology. The inkjet-printed sensor showed excellent reversibility and a great response of nearly 90% to saturated vapors of ethanol at RT, which could be attributed to the high surface-to-volume ratio of flowerlike-shape CuO nanoparticles. Sakthivel et al. [82] presented a FRT NH_3_ gas sensor fabricated by screen printing CuO nanorectangles material on PET substrate. The CuO nanorectangles were fabricated by a surfactant-free hydrothermal method with an average length and breadth of 950 and 450 nm, respectively. The RT sensor showed a meaningful response to 5 ppm of NH_3_ with response/recovery time of 90/120 s. In addition, the sensor exhibited tremendous stability over three months, performing its promising application prospects. As shown above, the solely reported CuO-based p-type pristine MOS-based FRT gas sensors did not perform great sensing properties compared to the n-type pristine MOS.

In brief, various morphologies of pristine MOS have been synthesized and transferred to flexible substrates by diverse methods for FRT gas sensors. These sensors have been widely used for the detection of various gases, and some excellent sensing performance has been achieved. In particular, one-dimensional (1D) nanostructure exhibit great potential to overcome high-temperature operation and low response due to their ultra-high surface-to-volume ratios and the large number of sites for adsorption of gas molecules, which is currently an effective strategy for achieving RT with pristine MOS. However, it should be noted that for most pristine MOS FRT gas sensors, their response at RT is much lower than that at higher operating temperatures, their response/recovery time are quite long, and sometimes these sensors cannot fully recover at RT after bending. To improve their sensing performance at RT, modification of pristine MOS materials is necessary, which will be discussed in the following chapters.

## Noble Metal Nanoparticles Modified MOS FRT Gas Sensors

Surface modification with noble metals refers to doping Pt, Pd, Au, Ag, and other noble metal nanoparticles on MOS to improve the surface activity and promote the catalytic decomposition of gas molecules, so as to make significant contributions to the better sensing performance [162–164]. Noble metal nanoparticles modification is considered to be a valuable strategy to improve the response and selectivity, reduce operating temperature and response/recovery time of the MOS gas sensors [165, 166].

### Mechanism of Noble Metal Modified MOS for Enhanced Gas Sensing Performance

Surface modification with noble metals can be carried out by chemical sensitization and electronic sensitization [167, 168]. Chemical sensitization increases the rate of chemical processes between target gas and chemisorbed oxygen through the catalytic action of noble metal nanoparticles, which is conducive to the easier migration of electrons, and thus enhance the performance of sensors. In chemical sensitization, the noble metal promoter activates the target gas by converting it into highly reactive molecules and accelerating it to spill over to the semiconductor surface, facilitating the catalytic oxidation [169], which is also referred to spill-over effect. Chemical sensitized noble metals do not directly affect the resistance of semiconductors and its sensing mechanism is the same as in the absence of doping. Electronic sensitization is the exchange of electrons between noble metal and MOS surface that directly affects the resistance of MOS [168]. The electronic sensitized noble metal forms stable noble metal oxides in air and are reduced to metal in a reducing gas atmosphere [170, 171]. As the oxidation state of noble metal varies with ambient atmosphere, the state of the electrons on the MOS surface changes accordingly. When the noble metal is oxidized, an electron depletion layer is established on the MOS surface, which directly affects the resistance of the semiconductor. On the other hand, as the noble metal oxide is reduced to metal, the electronic interaction with the MOS is disrupted, resulting in a decrease in the depth of electron depletion layer. These electronic sensitized noble metal oxides act as receptors for the target gas with much stronger electronic affinity than the adsorbed oxygen, enhancing the performance of the gas sensor [167].

### FRT Gas Sensors Based on Noble Metal Nanoparticles Modified MOS

**Fig. 3:**
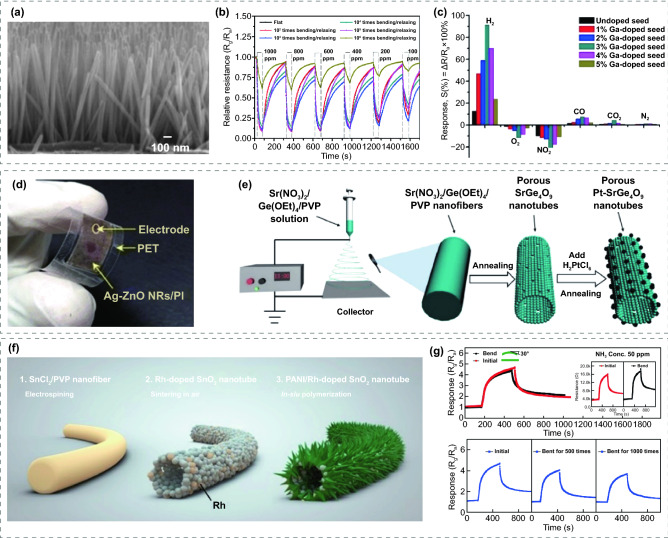
**a** Cross-sectional view of the Pd-ZnO nanorods/PI/PET sensor after 1000 times bending/relaxing test. **b** The response, reliability test of Pd-ZnO nanorods/PI/PET sensor. Reproduced with permission from Ref. [93]. Copyright (2013) Elsevier. **c** The selectivity of Pd–Ga-ZnO nanorods/PI sensor for various Ga-assisted seed layers conditions at 1000 ppm H_2_. Reproduced with permission from Ref. [91]. Copyright (2014) Elsevier. **d** The optical image of Ag-ZnO nanorods/PI sensor. Reproduced with permission from Ref. [99]. Copyright (2018) Springer Nature. **e** Schematic illustration of the fabrication process of Pt-SrGe_4_O_9_ nanotubes. Reproduced with permission from Ref. [80]. Copyright (2018) Springer Nature. **f** Schematic illustration of the fabrication process of PANI/Rh/SnO_2_. **g** Response of PANI/Rh/SnO_2_ sensors to 50 ppm NH_3_ at RT under bending test. Reproduced with permission from Ref. [172]. Copyright (2021) Elsevier

For many noble metal nanoparticles modified MOS flexible low-temperature gas sensors, 1D vertically well-aligned ZnO nanorods are often used as the sensitive layer due to the great inherent characteristics of ZnO and the simple hydrothermal synthesis method, for instance, Pd–ZnO nanorods/PI/PET [93], Pd–Ga–ZnO nanorods/PI [91] and Ag–ZnO nanorods/PI [99]. Chung et al. [93] presented a FRT H_2_ sensor based on the Pd-decorated ZnO nanorods. Figure 3a exhibited the ZnO nanorods remained vertically aligned after 1000 bending/relaxing test, exhibiting fabulous mechanical flexibility. As shown in Fig. 3b, the Pd-ZnO nanorods/PI/PET sensor showed a large response of 91.2% to 1000 ppm H_2_ at RT and great robustness with no significant degradation after 10^5^ bending cycles with a curvature angle of 90°. The modification of Pd nanoparticles not only enhances the sensor response, but also exhibits high selective absorption of H_2_. The authors attributed this enhancement to two-factors. One is that H_2_ molecules can be easily dissociated on the Pd surface: H_2_ + Pd $\leftrightarrow$ 2PdH_x_. Second, O_2_ in the ambient air can easily react with Pd nanoparticles and generate a weak-bonded state of PdO: 2Pd + O_2_ $\leftrightarrow$ 2PdO, which also can be dissociated and produce O_2_ easily. Furthermore, the same group [91] developed a FRT sensor based on perpendicularly aligned ZnO nanorods with Pd and Ga modifications. The Pd-3%Ga–ZnO nanorods/PI sensor showed excellent selective characteristics towards H_2_, as given in Fig. 3c. Beyond that, this sensor also exhibited a large response of 91% to 1000 ppm H_2_ at RT, which was improved six-fold compared with the undoped Ga-seed. More importantly, this sensor also performed excellent mechanical stability with no degradation after bending 10^5^ cycles, which might be related to its good crystallinity. Furthermore, the same group [99] also reported Ag nanoparticles modified ZnO nanorods gas sensor and its optical image is shown in Fig. 3d. The sensor can be activated under visible-light illumination: a large amount of photogenerated absorbed oxygen ions is generated owing to the coupling between Ag nanoparticles and ZnO nanorods, which results in an increase of surface charge density and an enhancement of sensing performance. This Ag–ZnO nanorods/PI sensor exhibited a linear response to C_2_H_2_ concentrations from 3 to 1000 ppm with the maximum response of 26.2 to 1000 ppm C_2_H_2_ at 130 °C. However, the sensor characteristics degraded obviously after bending 10^4^ cycles, which might be attributed to the fracture of the ZnO nanorods forest because of the excessive pressure.

In addition, 1D nanostructures are often fabricated through the controllable electrospinning method. Featured with regulated porosity, high surface-to-volume ratio, and tunable pore size, the electrospun 1D nanomaterials exhibit fabulous RT gas sensing properties. Moreover, modified with the proper noble metal catalyst, the 1D nanostructured MOS gas sensors show better RT sensing performance. A novel n-type wide-bandgap MOS sensing material, SrGe_4_O_9_ has been reported for the detection of NH_3_ at RT by Huang et al. [80]. Polycrystalline SrGe_4_O_9_ nanotubes were synthesized via a single-nozzle electrospinning process and Pt-modified SrGe_4_O_9_ nanotubes were prepared by annealing the mixture of SrGe_4_O_9_ and H_2_PtCl6 solution, as shown in Fig. 3e. The sensing materials were further assembled on a PET substrate to form a FRT sensor. The sensor exhibited a reliable detection of NH_3_ within the concentration of 1–500 ppm, a response of 7.08 to 100 ppm NH_3_ with fast response/recovery time of 17/16 s, excellent mechanical stability with a large bending angle of 150° and 10^3^ cycles of bending/relaxing test. Pt nanoparticles modified SrGe_4_O_9_ exhibited significantly enhanced response compared to the pristine SrGe_4_O_9_. The authors attributed the enhanced gas sensing performance to electronic sensitization and chemical sensitization. In electronic sensitization, a Schottky barrier was formed between SrGe_4_O_9_ and Pt, therefore, the electrons transferred from SrGe_4_O_9_ to Pt. In chemical sensitization, dissociation and adsorption of O_2_ molecules on the surface of SrGe_4_O_9_ were catalytically activated by Pt nanoparticles. The modification of noble metal nanoparticles can also enhance the sensing properties of metal oxide-based heterojunction. Rh-doped 1D hollow SnO_2_ nanotubes have also been reported for FRT NH_3_ sensors by Liu et al. [172]. As presented in Fig. 3f, they synthesized Rh-doped SnO_2_ hollow nanotubes by electrospinning and sintering. Subsequently, the PANI coated Rh-doped SnO_2_ hollow nanotubes was prepared by in situ polymerization and drop-coated on PET to form flexible sensors. Beyond that, Rh can be modified into the SnO_2_ lattice due to the similar ionic radii of Rh^3+^ and Sn^4+^, which contributed to the enhanced noble metal sensitization. The Rh modification not only facilitates the adsorption of NH_3_, but also promotes the decomposition of NH_3_ into highly reducing H and NH_2_, which can result in a rapid thinning the depletion region of SnO_2_, resulting in a fast response (113 s) and larger response values (13.6 to 100 ppm NH_3_). Moreover, as performed in Fig. 3g, the response of the sensor decreases only slightly at a bending angle of 30° and it is worth noting that the bending has no significant effect on the initial resistance of the sensor. Even after bending for 1,000 times, the sensor still possesses a reliable response, which means that the 1D nanostructure remain stable under bending situation.

In brief, modification with noble metals is also a superior scheme to enhance the response and selectivity due to the synergistic effect of chemical sensitization and electronic sensitization. Noble metal modification is generally effective in creating more defects, increasing the number of active sites, providing more oxygen species, and reducing the activation energy of the reaction between the gas molecules and adsorbed oxygens, thus accelerating the dynamic equilibrium between oxygen adsorption and desorption. Furthermore, some noble metals are specific for the detection of certain gases, which is beneficial for RT operation. For instance, Pd-modified MOS sensors exhibit a particularly large response to H_2_ due to the unique break-junction effect, while Rh-modified MOS sensors have a high response to NH_3_. Beyond that, the small size of noble metal nanoparticles does not affect the mechanical flexibility properties of the sensing material. All these features help to enhance the FRT gas sensing performance of MOS-based gas sensors.

## Organic Polymers Modified MOS FRT Gas Sensors

Organic conducting polymers-based gas sensors have attracted numerous interests due to their tunable electrical properties, simple fabrication, great stability, flexibility, environmental stability and RT operation [173–175]. However, the relatively low conductivity and the poor selectivity restrict the application of pristine conducting polymer-based sensors [174]. Therefore, coupling conducting polymers with other heterogeneous materials is a considerable strategy to enhance the sensing properties of the sensors [176–179]. Combing organic conducting polymers with MOS can complement the drawbacks of pristine MOS and organic conducting materials, especially the poor response and selectivity of pristine organic conducting polymers and the high operating temperature of MOS [180–182]. Organic conducting polymers including polyaniline (PANI), polypyrrole (PPy), polythiophene (PTh), poly (3,4-ethylenedioxythiophene) (PEDOT) and polyacetylene (PA) have been widely used in fabricating high-performance RT gas sensors [183]. However, only PANI is widely used for FRT gas sensors. PANI arrested the most interest because of its relatively high conductivity, ease of fabrication, RT operation, low cost, environmental stability, and friendliness [176, 184, 185].

### Mechanism of PANI Modified MOS for Enhanced Gas Sensing Performance

The gas sensing mechanism of pristine PANI has been widely investigated. Among them, the most commonly accepted mechanism was based on the PANI protonation/deprotonation process. In the doped emeraldine salt (ES) form, PANI is electrically conductive and, contrarily, in the dedoped emeraldine base (EB) form is insulating, where doping and dedoping can be carried out with acid or base, respectively [119]. The ability to switch between the conducting and insulating forms enables PANI responsive to acids/bases and reducing/oxidizing gases such as NH_3_, triethylamine (TEA), H_2_, NO_2_, and some VOCs.

The formation of heterojunction between PANI and MOS plays a significant role in the enhancement of the sensing properties. When PANI contacts MOS, the difference in Fermi energy levels leads to carrier transfer, forming a heterojunction and a narrow depletion region at their interface. When exposed to the target gas, PANI and the chemisorbed oxygen on the surface of MOS reacts rapidly, which modulates the width of the depletion region and rapidly affects the resistance of the sensing nanocomposites.

**Fig. 4:**
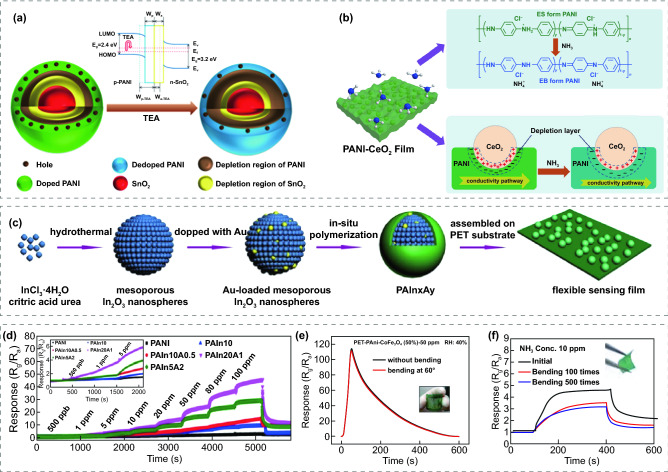
Schematic illustrations of the sensing mechanism of **a** PANI/SnO_2_ and **b** PANI-CeO_2_ for enhanced gas sensing performance. Reproduced with permission from Refs. [83, 110], Copyright (2018) Elsevier (2017) Elsevier. **c** The process flow for preparation of Au-In_2_O_3_@PANI sensors. **d** Transient response of the Au–In_2_O_3_@PANI sensors to 0.5–100 ppm NH_3_ at RT. Reproduced with permission from Ref. [79]. Copyright (2018) Elsevier. **e** Response of PANI-CoFe_2_O_4_ with a bending angle of 60° at 50 ppm of NH_3_. Reproduced with permission from Ref. [84]. Copyright (2021) MPDI. **f** Response of PANI @ porous nanospheres SnO_2_/Zn_2_SnO_4_ after 100 and 500 cycles of bending/relaxing test. Reproduced with permission from Ref. [186]. Copyright (2020) Elsevier

For instance, Quan et al. [110] synthesized network structures of PANI/SnO_2_ through in situ chemical oxidation polymerization. The mechanism for the enhanced sensing performance of the PANI/SnO_2_ composite was proposed. When p-type PANI is in contact with n-type SnO_2_, the electrons in SnO_2_ and holes in PANI will diffuse in opposite directions owing to their difference in Fermi energy levels. A p-n heterojunction and a narrow depletion region are formed at the interface of PANI and SnO_2_, as presented in Fig. 4a. In this process, at their interface, a hole depletion region is formed on the surface of PANI, while an electron depletion layer is formed on the surface of SnO_2_. When PANI/SnO_2_ is exposed to the atmosphere of the target gas TEA, on the one hand, the protons in the N^+^–H sites of PANI are drawn off and PANI is reduced from the conductive doped ES state to the insulating dedoped EB state, which leads to a decrease in the conductivity of the materials. On the other hand, the absorbed TEA molecules release electrons into the p-n heterojunction, which decrease the hole concentration of PANI and the electron concentration of SnO_2_, leading to a thickening of the hole depletion region and a thinning of the electron depletion region (Fig. 4a). Since the nanocomposite exhibits p-type semiconductor behavior, the conductivity of the composite decreases rapidly and result in enhanced sensing properties. The mechanisms of PANI modified MOS for enhanced gas sensing performance are mostly similar, and are illustrated based on the chemical state transition of PANI and the effective electron transfer in the heterojunction.

### FRT Gas Sensors Based on PANI Modified MOS

2D PANI nanosheets have been applied to construct the core–shell nanostructures of PANI and MOS nanocomposites, which can effectively enhance the sensing surface area and electrical conductivity of the bulk of sensing materials. Liu et al. [83] proposed a FRT trace-level NH_3_ sensor based on in situ self-assembled PANI-CeO_2_ nanomembranes on a PI substrate. The nanomembranes presented a core–shell nanostructure with a core of CeO_2_ nanoparticles and a shell of PANI nanosheet. The author suggested that CeO_2_ nanoparticles can influence the alignment of PANI and change the morphology of PANI shell. What’s more, an appealing discovery was observed that the synergistic oxidation of CeO_2_ and ammonium persulfate increases the protonation and oxidation of PANI, resulting in an enhanced = NH^+^–ratio, which would offer additional adsorption sites. Beyond that, the sensor performed a splendid gas sensing performance with large sensitivity of 262.7% to 50 ppm NH_3_, fabulous response-concentration linearity, great selectivity, ultralow detectable concentration of 16 ppb, and theoretical LOD of 0.274 ppb. In addition, there was no obvious decrease in response after 500 times bending, which might originate from the flexibility of polyaniline chains and the splendid adhesion and nano-mechanical properties of PANI-CeO_2_ nanomembranes. The authors ascribed the fabulous gas sensing performance to the synergetic benefits of the formation of p-n heterojunctions and the enhanced protonation degree of PANI, as presented in Fig. 4b.

PANI nanofibers [79, 81, 84, 87, 89, 110] and nanorods [85] usually have higher specific surface area and conductivity than the granular PANI owing to their 1D nanostructure, which facilitates electronic interactions between sensing material and target gas, and endows high response and low detection limit to FRT sensors. Li et al. [87] reported nanohybrids of PANI nanofibers and flowerlike WO_3_ nanoparticles, which was synthesized by an in situ chemical oxidation polymerization method. PANI grew on the flowerlike WO_3_ surface, forming a loose and porous nanostructure, which facilitates the adsorption and diffusion of NH_3_ molecules and promotes the modulation of the interfacial depletion region. Therefore, the fabricated sensors exhibited a high response of approximately 20.1 to 100 ppm NH_3_ at RT, which was 6 times larger than pristine PANI. What’s more, the sensor also showed rapid response/recovery time of 13/49 s, LOD of 500 ppb, fabulous moisture resistance, great selectivity, and excellent mechanical stability. The same group [79] presented a core–shell nanostructure with the core of Au-decorated In_2_O_3_ nanospheres and the shell of PANI nanofibers, which was synthesized by a facile hydrothermal and in situ chemical oxidation polymerization method (Fig. 4c). The fabricated nanohybrids were subsequently loaded on PET substrates to form flexible sensors. The sensing performance of sensors based on pristine PANI, In_2_O_3_@PANI, and Au–In_2_O_3_@PANI at RT were tested as presented in Fig. 4d. The Au–In_2_O_3_@PANI sensor showed a high response up to 46 to 100 ppm NH_3_ at RT, which is 14 and 4 times larger than the pristine PANI sensor and In_2_O_3_@PANI sensor, respectively. The fabulous sensing properties is contributed to the chemical sensitized effect of Au, the p-n heterojunction formed at the interface of In_2_O_3_ and PANI, and the improved protonation degree of PANI. Saleh et al. [84] presented a trace-level NH_3_ gas sensor composed of PANI nanofibers and CoFe_2_O_4_ nanoparticles on PET substrates by in situ chemical oxidation polymerization. The n-type CoFe_2_O_4_ nanoparticles were encapsulated in the p-type PANI, forming a p-n heterojunction at the PANI–CoFe_2_O_4_ interface. Therefore, the sensor exhibited great selectivity to NH_3_ and a significant response of 118.3% response towards 50 ppm for 24.3 s at RT. Notably, the sensor showed no response degradation while bending at 60° (Fig. 4e) and exhibited a trace-level detection limit of 25 ppb.

PANI with 1D morphology was also hybridized with Fe_2_O_3_ to acclimatize its structural properties, forming high-performance FRT gas sensors [81, 85, 89]. Bandgar et al. [81] displayed a RT NH_3_ sensor based on camphor sulfonic acid-doped PANI/*α*–Fe_2_O_3_ on PET substrates by in situ chemical oxidation polymerization method. Camphor sulfonic acid acted as a surfactant, contributing to the dispersion of *α*–Fe_2_O_3_ into PANI nanofibers matrix and the generation of active sites. This flexible sensor showed an ultrahigh selectivity towards NH_3_ compared to liquid petroleum gas (LPG), CH_3_OH, NO_2_, and C_2_H_5_OH. Zhu et al. [85] prepared a sea cucumber-shaped PANI/Fe_2_O_3_ nanocomposites by hydrothermal method, and assembled the nanocomposites on PET substrates. The sea cucumber-shaped PANI/Fe_2_O_3_ were assembled by PANI nanorods with small Fe_2_O_3_ nanoparticles attached. As the amount of precursor (FeCl_3_·6H_2_O) increases, the Fe_2_O_3_ nanoparticles become larger and the PANI nanorods become thicker. With a proper amount of precursor, the nanocomposites can have a porous nanostructure and a large surface-to-volume ratio, providing more active sites for efficient adsorption of NH_3_. Moreover, the sensor has great reproducibility, humidity resistance, and attain an excellent linear response to NH_3_ concentrations from 0.5 to 100 ppm at RT.

1D PANI nanofibers were also employed to modify heterostructured composites to form ternary compounds. Liu et al. [186] achieved rapid and selective detection of NH_3_ at RT by using in situ chemical oxidation polymerization method to assemble PANI@porous nanospheres SnO_2_/Zn_2_SnO_4_ nanocomposites on PET. The prepared sensor with the optimal PANI content exhibited a large response of 20.4 at RT, which is 2.6 times higher than that of the pristine PANI (7.8) and showed a detection limit as low as 500 ppb. However, the response decreased by 18.99% and 26.54% after 100 and 500 bending cycles, respectively, as shown in Fig. 4f. To find the explanation for the decrease in sensitivity, the morphology of the flexible sensor after 500 times of bending was observed through SEM. It was found that the location of the crease bending in the center of the sensor was damaged, leading to partial detachment and extrusion deformation of the nanocomposite. While the other parts mostly preserved the primitive morphology, which makes it possible to achieve reliable sensing for NH_3_ under bending conditions.

When PANI is used as the main component and MOS is used as a modifier of the sensing materials, the materials behave p-type characteristics acting like PANI. While constructing sensing materials with MOS as the main component and PANI as a modifier is also a possible strategy to fabricate high-performance gas sensors so that the sensing materials exhibit the desired n-type or p-type properties acting like MOS. However, the implementation of this strategy needs to depend on 0D PANI nanoparticles. Bai et al. [111] reported a FRT TEA gas sensor by loading *α*-MoO_3_ nanorods as a framework on a PET substrate and then covering the framework with 0D PANI nanoparticles through in situ chemical oxidation polymerization. The formation of p-n heterojunctions and the porous network morphology contribute significantly to the superior sensing performance. The sensor performed an excellent linear response to TEA at concentrations from 10 to 100 ppm at RT, and the LOD is theoretically calculated to be 0.55 ppm. What is worth noticing is that the negative influence of the relative humidity on the TEA sensors is negligible. The same group [86] also presented a heterostructure of 0D PANI nanoparticles modified with SnO_2_ nanofibers. The hybrid was further coated onto a PET substrate to develop a FRT NH_3_ gas sensor. The sensor reached a large response of 29 to 100 ppm NH_3_, which was 5 and 29 times larger than the pristine PANI or SnO_2_ based sensor, respectively.

Beyond that, PANI-modified MOS sensors also generally exhibit fabulous humidity resistance at RT. Some sensors even show increased response when the humidity increases, which is completely opposite to the conventional MOS-based gas sensors. This is due to the H_2_O molecules adsorbed on the surface of PANI acting as a proton source, which increases its doping level and the conductivity. Taking NH_3_ gas sensor, which is the main subject of this chapter, as an example, two reactions occur when exposed to NH_3_:

5 $${\text{NH}}_{{3}} \, + \,{\text{PANIH}}^{ + } \leftrightarrow {\mathbf{NH}}_{4}^{ + } + {\text{PANI}}$$

6 $${\text{NH}}_{{3}} \, + \,{\text{H}}_{{2}} {\text{O}}\, \leftrightarrow \,{\mathbf{NH}}_{4}^{ + } + {\text{OH}}^{ - }$$

Primarily, the NH_3_ molecules adsorbed on the surface of PANI take away protons from the -NH^+^- of PANI to form NH^4+^, and PANI changes from ES form to EB form, leading to an increase in the resistance of PANI. Furthermore, NH_3_ molecules dissolved in water generated OH^−^, which promoted the deprotonation of PANI. Under high humidity environment, these two reactions both facilitated the reaction degree of NH_3_ and enhanced the deprotonation of PANI, which contributed to the increased humidity resistance of the PANI-based RT sensor.

### FRT Gas Sensors Based on Other Organic Polymers Modified MOS

MOS nanoparticles have been incorporated in organic polymers to form flexible sensing materials in recent studies, which cannot only be used as the flexible substrate of gas sensors without constructing the extra substrate layer, but also can optimize the gas sensing performance. Mahmoud et al. [97] presented semi-conductive organic chitosan membrane based flexible H_2_S sensors. At first, WO_3_ nanoparticles were mixed with glycerin ionic liquid (IL) to form solutions. Then the homogenous solutions were cast into the organic chitosan sensing membrane. The presence of chitosan and glycerol increases the content of H-bonding, accelerating the electron accommodation and transfer. As a result, the fabricated gas sensors exhibited great sensing performance with fast response (13.6 s), a response of 2.75 to 200 ppm H_2_S, LOD of 15 ppm and excellent selectivity to H_2_S (compared to H_2_, NO_2_ and C_2_H_2_) at 40 °C. In addition, they also fabricated two other H_2_S gas sensors based on other organic materials [95, 96]. The flexible semiconductive polymeric matrix membranes [95] were prepared by mixing carboxymethyl cellulose (CMC) powders with 5% glycerin IL and 5 wt% CuO nanoparticles. The CMC/CuO/IL sensor showed a response of ~ 20% to 300 ppm H_2_S at low temperatures with a detection limit of 15 ppm. Before that, they also prepared novel flexible membranes [96] by embedding WO_3_ nanoparticles in PVA and glycerin IL polymeric solutions. More importantly, the conductivity of the mentioned membrane can be controlled by adjusting the proportion of glycerol. The environmental-friendly organic materials (chitosan, CMC, PVA) also provide a flexible matrix to accommodate IL and MOS nanoparticles.

Hydrogel is a 3D network-structured polymeric material formed by chemical or physical cross-linking and swelling in abundant water [187, 188]. Recently, novel ion-conductive hydrogel gas sensors have been proposed as one of the most promising flexible wearable sensors due to the tunable sensing properties, RT operation, excellent stretchability, transparency, biocompatibility, and facile synthesis process [189–191]. Wu et al. [192] synthesized SnO_2_-modified reduced graphene oxide hydrogels (SnO_2_/RGOH) with a 3D porous structure by simple hydrothermal process, and used a liquid crystal polymer as a flexible substrate to form flexible NO_2_ sensors with excellent mechanical bending tolerance. Compared with pristine RGOH, both sensitivity and LOD of SnO_2_/RGOH hydrogel were improved by one order of magnitude when operating at RT. The enhanced sensing performance of the hydrogel originated from the formation of p-n heterojunction at the interface of RGOH and SnO_2_, which facilitated the charge transfer. In addition, the large number of pores formed on 3D SnO_2_/RGOH hydrogel not only prompt the charge transfer through the charge hopping process, but also accelerate the gas diffusion through the pore filling effect. Besides, numerous oxygenated groups such as carboxyl and hydroxyl groups, which interact with NO_2_ molecules through hydrogen bonding, enhancing the adsorption capacity of NO_2_. It is worth noting that large mechanical deformations do not degrade the gas-sensitive performance of the 3D hydrogels due to the superior stretchability and self-healing properties, especially meet the need of flexible sensors. Technologies such as hybridization of various materials and 3D structure design will facilitate the development and practical application of MOS-based hydrogel gas sensors.

In brief, in this chapter, we introduced the sensing mechanism of PANI modified MOS for enhanced gas sensing performance, which is based on the protonation/deprotonation process of PANI and the enhanced sensitization of the heterojunction formed at their interface. Currently, 0D nanoparticles, 1D nanofibers, and 2D nanosheets of PANI have been employed to prepare high-performance FRT gas sensors of NH_3_ and TEA. Among them, 0D PANI nanoparticles attached on MOS exhibit excellent catalytic modification. 1D PANI nanofibers usually possess better RT sensing performance due to better electrical conductivity and higher surface-to-volume ratio. 2D PANI nanosheets performed excellent mechanical stability with no degradation under bending condition. In addition, PANI modified MOS sensing materials exhibited fabulous humidity resistance because the ES to EB transition process of PANI is accelerated in high humidity environment, expanding its application scope. Besides, we discussed flexible bulk materials with superior gas-sensitive properties based on other flexible organic polymers blended with IL and MOS. The promising MOS-based hydrogel FRT gas sensor was also briefly presented.

## Carbon-Based Materials Modified MOS FRT Gas Sensors

1D carbon nanotubes (CNTs) and 2D graphene derivatives are emerging materials for gas sensing owing to their high sensitivity, large surface-to-volume ratio, and abundant adsorption sites [120, 193, 194]. Nevertheless, target gas molecules are adsorbed on the carbon-based materials mainly depend on weak van der Waals forces, which restrict the high sensing performance of sensors [149]. To overcome the shortcomings, hybridization of carbon-based materials with MOS has been introduced, and this complementary hybridization not only complements the drawbacks of single components, but also introduces promising advantages [195, 196]. Recently, several reviews have reported carbon-based materials modified MOS gas sensors with enhanced sensing performance [197–199]. The results suggest that the carbon-based material/MOS heterostructure and the unique morphology of the composites contribute to the enhancement of the high-performance gas sensors [200–202].

### Mechanism of Carbon-Based Materials Modified MOS for Enhanced Gas Sensing Performance

In carbon-based materials modified MOS, the main role of MOS is to detect the target gas molecules, while carbon-based materials can serve as great acceptors for injected electrons owing to their high electrical conductivity, large surface area and abundant adsorption sites. Although little is known about the gas sensing mechanism of these hybrids, the data reported so far suggest that their response mechanism is based on the modulation of the heterojunction between the MOS and the carbon-based material.

In general, both CNTs and graphene derivatives exhibit p-type properties. When a carbon-based material is in contact with MOS, a p–n junction or a p–p junction is formed at the interface, depending on whether the MOS material is n-type or p-type. When exposed to an oxidizing gas atmosphere, the oxidizing gas extracts electrons from the heterojunction, the interfacial potential barrier and the thickness of the depletion region increases, which amplifies the increase in resistance. However, when exposed to a reducing gas atmosphere, the reducing gas releases electrons to the heterojunction, the interfacial potential barrier and the thickness of the depletion region decreases, leads to the sharp decrease of resistance. Beyond that, the heterojunctions can also enhance oxygen adsorption, thus forming a large number of oxygen vacancies on the surface of nanocomposites, providing extra active sites for sensing reactions.

Furthermore, during the synthesis process, the carbon-based material can modulate the size and morphology of the metal oxide. Besides, the carbon-based materials increase the electrical conductivity of the composites, which can transfer the electrons of sensing reactions to the metal electrodes rapidly. What’s more, carbon-based materials contains a large number of mesopores, which facilitate the adsorption and desorption of gas molecules. These will improve the sensitivity and response speed of carbon-based materials modified MOS gas sensors.

### FRT Gas Sensors Based on CNTs Modified MOS

**Fig. 5:**
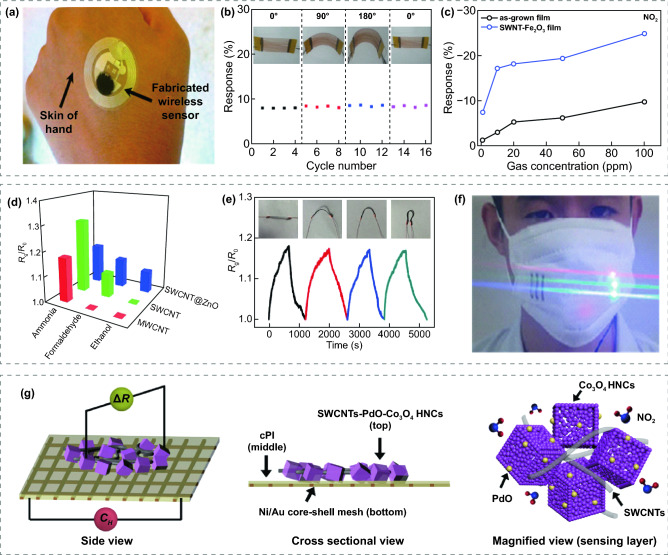
**a** The optical image of manufactured wireless RFID sensor. Reproduced with permission from Ref. [112]. Copyright (2016) Elsevier. **b** Response of Fe_2_O_3_-SWCNTs sensor under large-degree deformation. **c** Response of SWCNTs and Fe_2_O_3_-SWCNTs sensor towards NO_2_ (1, 10, 20, 50, and 100 ppm). Reproduced with permission from Ref. [68]. Copyright **(**2017**)** Elsevier. **d** Response of the SWCNT, MWCNT and SWCNTs@ZnO nylon sensors to 500 ppm NH_3_, HCHO and C_2_H_5_OH at RT. **e** Response of the nylon-based sensor under large-degree deformation. **f** The optical image of a multifunctional mask with integrated NH_3_, HCHO and C_2_H_5_OH sensors. Reproduced with permission from Ref. [100]. Copyright **(**2018**)** Springer Nature. **g** Schematic illustrations of SWCNT/PdO/Co_3_O_4_ on flexible Ni/Au-PI film. Reproduced with permission from Ref. [74]. Copyright **(**2017**)** American Chemical Society

CNTs is a widely used carbon-based material for gas sensing owing to its high specific surface area, fabulous electrical conductivity, and great flexibility characteristics [203]. In addition, CNTs provide an effective conductive pathway for electron transport, further improving the response value and response rate of the CNTs/MOS sensors [204]. Single-walled carbon nanotubes (SWCNTs) are cylindrical nanotubes rolled up by honeycomb lattice carbon sheets. The hybrid of SWCNTs and MOS exhibits great sensing performance, especially low detection limits. Asad et al. [112] fabricated flower-shaped CuO-SWCNTs through a hydrothermal method and then the obtained materials were drop-casted on the RFID. As shown in Fig. 5a, the obtained wireless RFID sensors can be perfectly attached to the skin, revealing their applications for healthcare and environmental monitoring. Beyond that, the fabricated sensor not only selectively detected H_2_S with an ultralow LOD of 100 ppb at RT, but also showed a response of 35% to 1 ppm H_2_S with 7 s response time. Hua et al. [68] prepared SWCNT films by floating catalytic chemical vapor deposition with ferrocene as the catalyst, and then twig-like Fe_2_O_3_ nanoparticles were obtained by a simple annealing process, which are attached on the surface of SWCNT bundles. The as-grown Fe_2_O_3_-SWCNTs were then transferred onto PP plastic substrates to form flexible gas sensors, and showed great mechanical robustness after large-degree deformation, as shown in Fig. 5b. The fabricated Fe_2_O_3_–SWCNTs sensors obtained an enhanced response towards NO_2_, as shown in Fig. 5c, and exhibited a significant response towards H_2_S, while the pure SWCNTs sensor failed to detect H_2_S. The improved sensing performance is ascribed to the presence of Fe_2_O_3_ nanoparticles, which accelerates electron transport and promotes the electron–hole recombination. Gao et al. [100] prepared flexible nylon fiber gas sensors with SWCNTs, multi-walled carbon nanotubes (MWCNTs) and ZnO quantum dot modified SWCNTs as sensing materials. Remarkably, as shown in Fig. 5d, SWCNTs@ZnO exhibited a significant response to ethanol while the SWCNTs and MWCNTs did not, which is contributed to the effect of ZnO quantum dots. The presence of ZnO quantum dots reduces the defects of SWCNTs and decreases the charge density, creating a high conductive state serves as a reduction of the gating effect at the hybrid interface. In addition, the flexible gas sensor exhibited good mechanical bending ability and robustness, as shown in Fig. 5e. Robustness test towards washing treatment was also conducted by washing the nylon sensors for different times and the sensors showed no significant degradation. More importantly, the fabricated flexible sensors were further integrated into face masks, selectively detected NH_3_, HCHO and C_2_H_5_OH by distinguishing whether the corresponding LEDs are lighted or not, as shown in Fig. 5f.

SWCNTs can also be easily blended with other sensing materials to synthesize multivariate composites. Furthermore, noble metal nanoparticles can be easily incorporated into various sensing materials by simple synthesized methods such as hydrothermal, wet impregnation, and physical sputtering methods. Therefore, noble metal nanoparticles are often used to fabricate ternary or even multivariate composites to enhance the performance of sensors [74, 79, 205–207]. Kim et al. [74] fabricated SWCNTs decorated PdO/Co_3_O_4_ hollow nanocubes for NO_2_ sensors. PdO–Co_3_O_4_ hollow nanocubes were synthesized by calcining of Pd-infiltrated Co-based ZIF-67. Subsequently, SWCNTs were mixed with PdO-Co_3_O_4_ hollow nanocubes dispersed in ethanol to help electrically bridging multiple PdO/Co_3_O_4_, which can increase the baseline electrical conductivity. With the Pd nanoparticles modification, the hole accumulation layer on the Co_3_O_4_ nanocubes gets thicker and accelerates the redox reaction with NO_2_ through the electronic sensitization. As shown in Fig. 5g, the SWCNTs/PdO/Co_3_O_4_ composite was further integrated on the Ni/Au-PI film to construct FRT gas sensors. Moreover, the sensor exhibited reliable detection of NO_2_ at RT with a high response of 27.33% at 20 ppm and a LOD of 1 ppm.

**Fig. 6:**
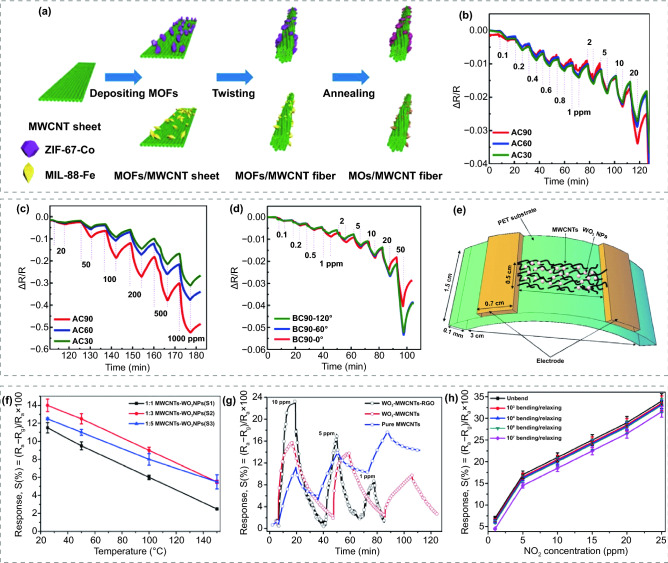
**a** The process flow for preparation of MOFs modified MWCNT fibers. Transient response of Co_3_O_4_/MWCNT hybrid fibers to **b** 0.1–20 ppm **c** 20–1000 ppm NO_2_ at RT. **d** Transient response of the Co_3_O_4_/MWCNT hybrid fibers to 0.1–50 ppm NO_2_ at 0°, 60°, and 120°. Reproduced with permission from Ref. [64]. Copyright (2018) American Chemical Society. **e** Schematic diagram of WO_3_/MWCNT-based sensor. **f** Response of WO_3_/MWCNT-based sensor as a function of operating temperature. Reproduced with permission from Ref. [73]. Copyright (2015) Elsevier. **g** Transient response of pure MWCNTs, WO_3_/MWCNTs and WO_3_/MWCNT/RGO. **h** Response of WO_3_/MWCNT-based sensor after different cycles of bending/relaxing tests. Reproduced with permission from Ref. [72]. Copyright (2016) Elsevier

MWCNTs are concentric graphene rolled up with diameters on the order of hundreds of nanometers. Unlike the disordered arrangement of SWCNTs, MWCNTs are ordered and exhibit good electrical properties with enhanced charge migration in the direction of the arrangement. The reported FRT sensors based on the hybrid of MWCNTs and MOS exhibit ultralow detection limits. Rui et al. [64] used MOFs as precursors for MOS and aligned MWCNT nanofibers as the conductivity path for NO_2_ sensing. First, they introduced ZIF–67–Co into the stacked MWCNT nanosheets by drop-casting method. Then, the MOFs modified MWCNT nanosheets were twisted and annealed in air to form Co_3_O_4_/MWCNT hybrid fibers, as shown in Fig. 6a. The hybrid fiber was further sewn into commercially textile fabrics without damage. The manufactured textile fabrics NO_2_ sensor performed a broad detection range from 0.1 to 1000 ppm at RT, as given in Fig. 6b, c. The porous MWCNT fibers help facilitating electron interactions and promoting electron transfer reactions. As depicted in Fig. 6d, the hybrid fiber sensor performed even better response when bent into different angles, which might be ascribed to the promotion of electron transfer along the straightened MWCNT fiber under tension. The fabricated smart textiles are also equipped with energy storage functions, demonstrating potential applications for integrated wearable devices. Yaqoob et al. [73] mixed the prepared WO_3_ nanoparticles and MWCNTs with the assistance of α-terpineol, and then fabricated flexible sensors through gel casting on PET substrates, as shown in Fig. 6e. The sensor displays an interesting phenomenon that the response decreases as the operating temperature increases (Fig. 6f), which can be ascribed to the elimination or decline of the nano-Schottky barrier or p-n heterojunction between WO_3_ and MWCNTs at higher temperature. This interesting phenomenon is contrary to the properties of MOS and informative to reduce the working temperature of MOS-based sensors. Furthermore, they mixed reduced graphene oxide (RGO) powders into the mixture and formed a WO_3_/MWCNT/RGO-based sensor on a PI/PET substrate [72]. The Brunauer, Emmett and Teller analysis [208] illustrated that the specific surface area of WO_3_/MWCNT/RGO was larger than WO_3_/MWCNT. In addition, as shown in Fig. 6g, both WO_3_/MWCNT/RGO-based and WO_3_/MWCNT-based sensors exhibit a large response to NO_2_. The presence of WO_3_ nanoparticles not only forms a p-n heterojunction, but also provides an efficient electron transfer pathway. As for the modification of RGO, it can effectively increase the specific surface area and facilitate the adsorption–desorption kinetics to achieve better gas sensing properties. What’s more, the WO_3_/MWCNT/RGO-based and WO_3_/MWCNT-based sensors showed low detection limit of 1 and 0.1 ppm, respectively. However, they both suffered from the long response times compared with other types of MOS-based sensors. Figure 6h represents the response of WO_3_/MWCNT/RGO-based sensor under different bending-relaxing cycles. No remarkable degradation after 10^6^ repeated cycles, which can be explained by the fabulous flexible properties of MWCNTs and RGO.

### FRT Gas Sensors Based on Graphene Derivatives Modified MOS

**Fig. 7:**
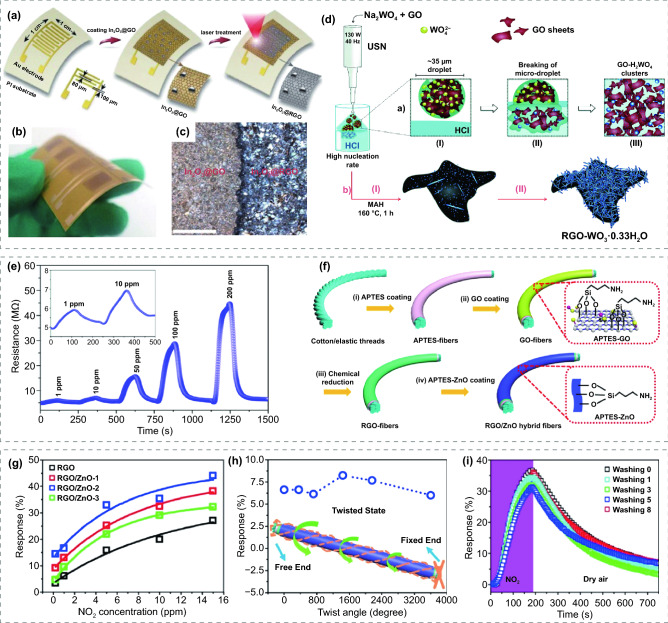
**a** Schematic illustration of the fabrication process for In_2_O_3_@RGO sensors. **b** The optical image of the 2 × 4 In_2_O_3_@RGO sensor array. **c** The confocal laser scanning microscopy images of the border of GO and RGO. Reproduced with permission from Ref. [65]. Copyright (2018) Elsevier. **d** Schematic illustration of the fabrication process for RGO/WO_3_·0.33H_2_O hybrids. **e** Transient resistance of the RGO/WO_3_·0.33H_2_O hybrids. Reproduced with permission from Ref. [108]. Copyright (2018) The Royal Society of Chemistry. **f** The process flow for preparation of RGO/ZnO hybrid threads. **g** Transient response of RGO and RGO/ZnO to different NO_2_ concentration. **h** Response of RGO/ZnO sensors under different twist angles in 2 ppm NO_2_ (0°, 360°, 720°, 1440°, 2160°, and 3,600°). **i** Response versus time plot of the RGO/ZnO sensors under different washing times. Reproduced with permission from Ref. [54]. Copyright (2019) American Chemical Society

Being one of the brightest promising materials, graphene attracted the extensive enthusiasm of researchers in the field of gas sensing owing to its outstanding overall characteristics, such as single atom thickness, 2D layered nanostructures, high Young's modulus of elasticity, room-temperature stability, large surface-to-volume ratio, and excellent thermal and electrical conductivity [209–211]. The synergetic effect between MOS and graphene for the enhanced selectivity and sensitivity of gas sensors have been well demonstrated [118, 212]. Goutham et al. [213] fabricated a FRT transparent LPG sensor based on CdO/graphene hybrid. The presence of graphene reduces the aggregation of CdO nanoparticles, increases the surface-to-volume ratio and accelerates electron transfer by providing more conducting channels. Graphene derivatives are also promising candidates for the exploration of high-performance carbon-based gas sensors. Compared to pristine graphene and other graphene derivatives, RGO is prevailing owing to its fabulous characteristics such as large specific surface-to-volume ratio, great sensitivity, chemical and mechanical stability, and high carrier mobility. In addition to the synergetic benefits of MOS and RGO, the synthesis method of RGO is one of the most significant aspects affecting the properties of the RGO/MOS-based sensor. You et al. [65] assembled a In_2_O_3_@RGO-based 2 × 4 NO_2_ flexible sensor array by direct laser writing (DLW) process. The schematic diagram of the manufacture of the In_2_O_3_@RGO FRT sensors and the optical photograph of the sensor array is shown in Fig. 7a, b. Confocal laser scanning was conducted on the border of GO and RGO, and the microscopy pictures is presented in Fig. 7c, exhibiting sharped edges that reveal the high accuracy of DLW treatment. What’s more, the surface and edges of GO sheets contain numerous oxygen containing groups (OCGs), which makes GO insulating. After DLW treatment, the resistance of In_2_O_3_@GO decreased from ∼7.6 × 10^8^ to ~ 230 Ω due to the removal of OCGs. More importantly, DLW can also effectively promote the photoreduction of GO, allowing In_2_O_3_@RGO to be patterned on flexible substrates. The sensor exhibits a linear response to trace-level NO_2_ over the concentration of 50–1000 ppb at RT with large response (31.6% to 1 ppm NO_2_) and excellent selectivity. However, the sensor performed poorly regards to response/recovery time (4.2/13.3 min). The great sensing performance could be contributed to the recovery conductivity of GO, the p-n heterojunctions at the In_2_O_3_/RGO interface, and the formation of nanopores on GO sheets during DLW treatment owing to the escape of the OCGs through CO_2_, H_2_O, CO, etc.

It is known that WO_3_ has several crystalline hydrates such as WO_3_·2H_2_O, WO_3_·H_2_O and WO_3_·0.33H_2_O. Among them, WO_3_·0.33H_2_O exhibits the best sensing performance at RT [214]. Perfecto et al. [108] fabricated a FRT isopropanol sensor based on the composite of RGO and WO_3_·0.33H_2_O nanoneedles. The RGO/WO_3_·0.33H_2_O composite was obtained by ultrasonic spray nozzle (USN) and microwave-assisted hydrothermal (MAH), as shown in Fig. 7d. The agglomerates containing H_2_WO_4_ with GO sheets can be converted to GO-H_2_WO_4_ clusters in a high nucleation rate by USN method, and the MAH method helps to facilitate the reduction of GO and form WO_3_ nanoneedles on RGO sheets. As illustrated in Fig. 7e, the sensor exhibited a large and linear response to isopropanol at concentrations of 10–100 ppm. In addition, the response time for different isopropanol concentrations is relatively short, ranging from 60 to 90 s. Li et al. [54] prepared a FRT NO_2_ gas sensor with commercial flexible cotton/elastic threads substrates and RGO/ZnO nanosheets as a sensing material to fabricate conductive threads. Figure 7f demonstrated the manufacturing flow for RGO/ZnO composite threads. The introduction of APTES coating helps to strengthen the adhesion of GO coating on threads. After coating the GO film, a further chemical reduction reaction was performed to form RGO-threads. The RGO-threads were further immersed in an aqueous APTES-ZnO solution and subsequently annealed to form RGO/ZnO hybrid threads. Figure 7g exhibits the response of RGO/ZnO hybrid threads to NO_2_, which was significantly enhanced over pristine RGO sensor. Furthermore, the sensor exhibited great long-term stability (84 days) and low detection limit (200 ppb). Beyond that, the fabricated sensing threads exhibited superior mechanical and washing durability, as shown in Fig. 7h, i. With cotton/elastic threads as the flexible substrate, the sensor can even be stretched, twisted, and washed, providing a new perspective on wearable E-textiles for practical multifunctional applications.

**Fig. 8:**
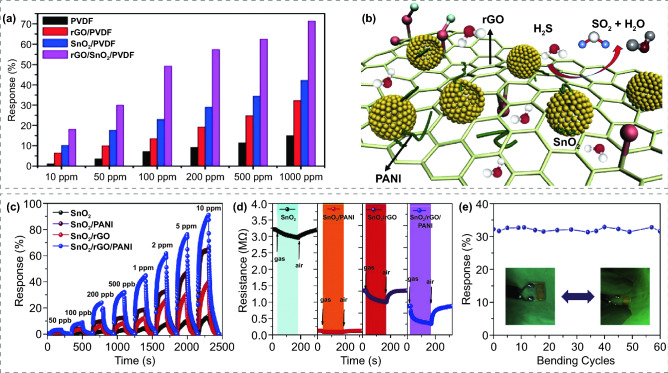
**a** Response comparison among the pristine PVDF, RGO/PVDF, SnO_2_/PVDF, and RGO/SnO_2_/PVDF composites. Reproduced with permission from Ref. [94]. Copyright (2020) Springer Nature. **b** The sensing mechanism diagram of SnO_2_/RGO/PANI nanocomposites towards H_2_S. **c** Response comparison among SnO_2_, SnO_2_/PANI, SnO_2_/rGO and SnO_2_/rGO/PANI. **d** Transient resistance of SnO_2_, SnO_2_/PANI, SnO_2_/RGO and SnO_2_/RGO/PANI towards 2 ppm H_2_S. **e** The response of SnO_2_/RGO/PANI hybrid to 500 ppb H_2_S after bending test. Reproduced with permission from Ref. [98]. Copyright (2019) Elsevier

RGO sheets are also excellent matrices for constructing multivariate composites. The synergistic effect of RGO and other multiple nanomaterials provides unprecedented possibilities for innovative nanostructures and high performance of gas sensors. Punetha et al. [94] prepared optimized ternary nanocomposites based on RGO, SnO_2_, and polyvinylidene fluoride (PVDF) by a simple hot press method. The proposed ternary nanocomposite-based flexible sensor showed great response of 49.2% to 100 ppm H_2_ at RT, which is 2 and 3.5 times larger than SnO_2_/PVDF and RGO/PVDF sensors, respectively, as shown in Fig. 8a. In addition, the RGO/SnO_2_/PVDF nanocomposite showed excellent stability with almost no response degradation after being tested for a month and presented a detection limit up to 500 ppb. Zhang et al. [98] synthesized sub-ppb level FRT H_2_S sensors based on SnO_2_/RGO/PANI nanocomposites. The ternary composite is closely packed together in the form of a porous network structure, which helps accelerating the absorption and desorption of H_2_S, as shown in Fig. 8b. The fabricated sensor showed fabulous sensing properties with high sensitivity (60.11% to 2 ppm H_2_S), response/recovery time (82/78 s), stable repeatability, and long-term stability. Figure 8c illustrates that the SnO_2_/RGO/PANI hybrid exhibited significantly improved response compared to pristine SnO_2_ as well as the hybrid of SnO_2_/RGO and SnO_2_/PANI, demonstrating the superiority of the ternary compound. The dynamic resistance transition of the sensors towards 2 ppm H_2_S also indicated the effective modulation of the baseline resistance by SnO_2_/RGO/PANI composite (Fig. 8d), which contributes to the expansion of the electronic pathway. Beyond that, the response of SnO_2_/RGO/PANI hybrid showed no degradation after bending test (Fig. 8e). The reported ternary composite-based gas sensors reveal that ternary composite might help exploring a new route to develop novel FRT sensors with high performance, but the mechanical properties still need to be further studied to prevent the generation of fractures.

In brief, this chapter presented the sensing mechanism of carbon-based material modified MOS sensors. Carbon-based materials are beneficial to promote electron transfer and oxygen adsorption/desorption due to their high conductivity, large surface area, and abundant adsorption sites. In addition, the heterojunction formed between the carbon-based material and MOS facilitates the rapid modulation of resistance and lower operating temperature. Moreover, both 1D CNTs and 2D graphene derivatives consist of a honeycomb-like lattice network of carbon atoms that remain resistant to fracture under bending. Besides, the carbon-based materials provide more conductive channels that allow carbon-based material modified MOS sensors to remain responsive after bending test. The potential of RGO nanosheets to construct excellent matrices of multivariate composites is also presented. The synergistic effect of RGO, MOS, and tertiary materials such as noble metals and PANI, endows to achieve incredible gas sensing performance. To sum up, the nanocomposites of MOS with carbon nanomaterials have much shorter response/recovery times, lower operating temperature, better mechanical flexibility, and lower detection limits than pristine MOS, although the increase in response values obtained with these nanocomposites might be less pronounced.

## TMDCs Materials Modified MOS FRT Gas Sensors

Recently, gas sensing semiconducting 2D TMDCs have aroused growing interest owing to their attractive properties that stem from their narrow band gap, large surface area provided by the sheet-like nanostructure, and unique surface and quantum effects [215–217]. In addition, TMDCs has a lamellar structure with weak van der Waals forces between adjacent layers, which facilitates gas adsorption. And due to the unique electrical properties, TMDCs can operate at lower operating temperatures, reducing the overall power consumption and eliminating the need for external heating. It also has superior inherent electrical conductivity, thermal stability, and oxidative stability [218, 219]. However, the pristine TMDC-based gas sensors exhibit low and slow response, inadequate recovery and poor selectivity [118, 220]. Therefore, promising 2D heterostructures combined with TMDC and MOS have been proposed due to their integrated advantages of both components. However, there are few papers proposed to fabricate the FRT gas sensor based on TMDC/MOS composites.

**Fig. 9:**
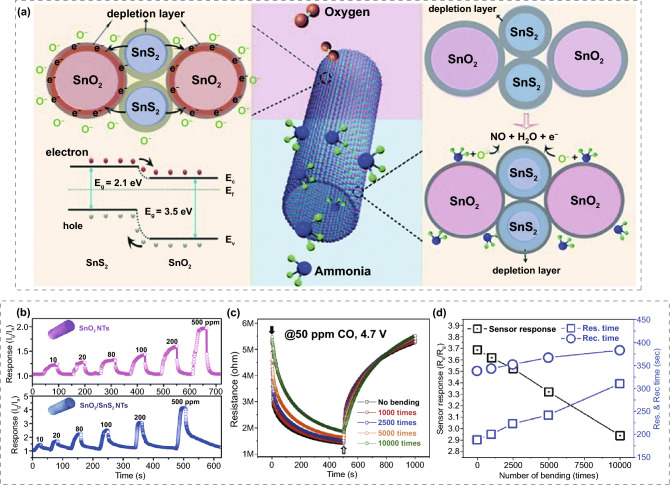
**a** The sensing mechanism diagram of SnO_2_/SnS_2_ nanotube in air and NH_3_ atmosphere. **b** Transient response of the pristine SnO_2_ and SnO_2_/SnS_2_ to 10–500 ppm NH_3_ at RT. Reproduced with permission from Ref. [88]. Copyright (2017) The Royal Society of Chemistry. **c** Dynamic resistance of Au/SnO_2_/WS_2_ to 50 ppm CO after different cycles of bending tests. **d** Response values and response/recovery time of Au/SnO_2_/WS_2_ to 50 ppm CO after different cycles of bending tests. Reproduced with permission from Ref. [221]. Copyright (2021) Elsevier

Li et al. [88] used electrospun synthesized SnO_2_ nanotubes as a backbone and prepared SnO_2_/SnS_2_ nanotubes using an in situ hydrothermal method, and then spun-coated the samples on PET substrates to form flexible sensors. Figure 9a illustrates the mechanism of SnO_2_/SnS_2_ nanotube in air and NH_3_ atmosphere. The work function of SnO_2_ is smaller than SnS_2_ so that the electrons are transferred from SnS_2_ to SnO_2_. Electron depletion region and accumulation region were established on the surface of SnS_2_ and SnO_2_, respectively. When the sensor is in NH_3_ atmosphere, gas molecules interact with O^−^, reducing the thickness of depletion region and the resistance. The heterojunction effect of SnO_2_ and SnS_2_ contributes to enhance the sensing performance. As presented in Fig. 9b, the SnO_2_/SnS_2_ nanotubes sensors showed a response of 2.48 to 100 ppm NH_3_, which was almost two times higher than the pristine SnO_2_ nanotubes sensors. Beyond that, the sensor presented a relatively rapid response/recovery time of 21/110 s, LOD of 1 ppm, outstanding selectivity. More importantly, the flexible SnO_2_/SnS_2_ nanotubes exhibited fabulous mechanically flexibility with no response decrease after bending 3,000 times or under 150° bended angle. The fabulous FRT gas sensing performance is attributed to the high aspect ratio of nanotubes and the effect of SnS_2_ modification. Kim et al. [221] implemented Au–SnO_2_–co-modified WS_2_ nanosheets on PI substrate. The sensor exhibited the highest response of 3.687 to 50 ppm CO at an optimum operating voltage of 4.7 V. More importantly, the optimized TMDCs-based gas sensor exhibited fabulous sensing performance under tilting, bending, and stretching conditions. As presented in Fig. 9c, d, after 10^4^ times bending or tilting test, the flexible sensors still exhibited a remarkable and reliable response, which is largely derived from the high carrier mobility and the layered nanostructure of WS_2_. Furthermore, for the ternary compound of Au–SnO_2_–co–modified WS_2_ nanosheets, Schottky junctions form at the interface of Au and SnO_2_ as well as Au and WS_2_, while heterojunctions form at the interfaces of SnO_2_–WS_2_ and Au/SnO_2_/WS_2_, providing a large number of easily modulated heterostructures. When exposed to CO atmosphere, the release of electrons can effectively modulate the potential barrier height of these heterojunctions for rapid and high response. In addition, the self-heating effect endows the sensor with excellent RT properties. When applying a voltage, the intrinsic resistance of the WS_2_ nanosheets and the numerous heterojunctions are great sources of Joule heating, enabling the sensors without additional heaters. Kang et al. [222] prepared MoS_2_ on SiO_2_ by CVD and transferred to a flexible PET substrate, after which 2-nm-thick SnO_2_ was deposited on its surface by e-beam evaporation for NO_2_ detection. Under visible light activation, unlike the pristine MoS_2_, which showed little variation in response to different concentrations of NO_2_, the functionalized MoS_2_ showed responses of 2.43, 5.84, and 7.57 at RT when exposed to 3, 6, and 9 ppm NO_2_, respectively. This suggests that phototransfer of electrons at the interface heterojunction between MoS_2_ and SnO_2_ plays a crucial role in the detection of target gas.

In brief, 2D TMDCs are a novel gas-sensitive material due to their unique surface effect and low operating temperature. TMDCs modified MOS FRT gas sensors sufficiently exploit the superior mechanical flexible lamellar nanostructure of TMDCs and substantially increase the slow and low response of pristine TMDCs by modulating the heterojunction between MOS and TMDCs. Although there are few papers focus on the preparation of TMDC modified MOS FRT gas sensors, its tremendous mechanical flexibility and the low operating temperature make it a promising material for FRT sensors.

## Light-illuminated MOS FRT Gas Sensors

### Mechanism of Light-Illuminated MOS for Enhanced Gas Sensing Performance

Light illumination is also a superior strategy to enhance the gas sensing properties [223, 224], decreasing the operating temperature of MOS gas sensor [77, 225, 226], and providing a distinctive pathway to achieve FRT MOS gas sensors [227]. When the device is irradiated by light with a certain wavelength, if the photon energy is larger than the MOS bandgap, photogenerated electron–hole pairs will be generated, as listed in Eq. 7. The photo-generated holes will interact with the chemisorbed oxygen ions on the MOS surface to release oxygen, as listed in Eq. 8, leading to the decrease of the depletion region width and the bulk resistance. In other respects, the photo-generated electrons will interact with ambient oxygen molecules to form photo-induced oxygen ions, as listed in Eq. 9. Unlike the strong adhesion of chemisorbed oxygen ions on the MOS surface, the photo-induced oxygen ions have weak bonding with MOS and thus react easily with target gas, resulting in an enhanced gas sensing response at RT.

7 $$\mathrm{hv}\to {\mathrm{h}}^{+}(\mathrm{hv})+{\mathrm{e}}^{-}(\mathrm{hv})$$

8 $${\mathrm{h}}^{+}(\mathrm{hv})+{\mathrm{O}}_{2}^{-}\to {\mathrm{O}}_{2}$$

9 $${\mathrm{e}}^{-}(\mathrm{hv})+{\mathrm{O}}_{2}\to {\mathrm{O}}_{2}^{-}(\mathrm{hv})$$

### FRT Gas Sensors Based on Light-Illuminated MOS

Several FRT gas sensors under light-illuminated have been reported [66, 70, 75, 99, 102, 105, 160]. The pristine ZnO is a direct bandgap semiconductor with a bandgap of 3.35 eV [228], thus it can be activated by UV-light and generate photo-generated electron–hole pairs to effectively lower the operating temperature. Zheng et al. [102] presented a UV-activated flexible ethanol sensor through depositing ZnO nanoparticles on a PET-ITO substrate. Under UV light illumination, the sensor exhibited a large response of 2.2 to 800 ppm ethanol at RT. In addition, using flexible transparent PET-ITO as a substrate, the sensor has a transmission rate of over 62% in the visible range of 400–800 nm. As another example, Jacobs et al. [160] reported a flexible UV-irradiation RT ZnO sensor made of low-cost, biologically-derived ZnS nanoparticles that can be used to effectively detect and discriminate between O_2_ and H_2_O.

**Fig. 10:**
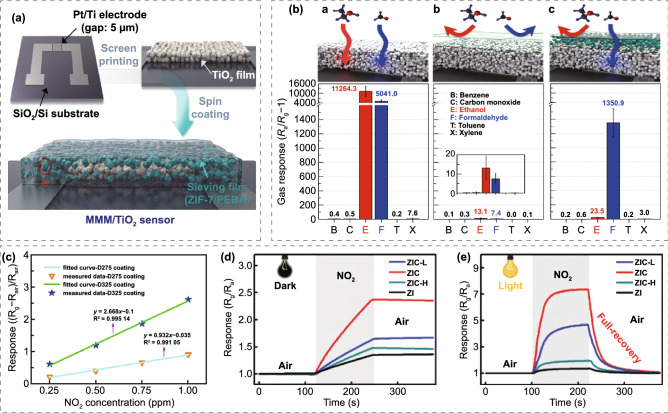
**a** The process flow for preparation of ZIF-7/PEBA coated TiO_2_ sensors. **b** Gas responses of bare TiO_2_^a^, pure PEBA/TiO_2_^b^, and 5MMM/TiO_2_^c^ sensors exposed to different gas with concentration of 5 ppm at RT under UV-illumination. Reproduced with permission from Ref. [105]. Copyright (2021) Springer Nature. **c** The linear responses of ZnO_1−x_ based sensors to 0.25-1 ppm NO_2_ under white-light illumination. Reproduced with permission from Ref. [70]. Copyright (2017) Elsevier. Transient response of ZI, ZIC, ZIC-L (lower g-C_3_N_4_ content), ZIC-H (higher g-C_3_N_4_ content) sensors to 1 ppm NO_2_ at RT **d** in dark, **e** under visible-light illumination. Reproduced with permission from Ref. [66]. Copyright (2021) Wiley–VCH

Photoactivation and molecular sieving can also be combined to achieve exclusive selectivity and large response at RT. Jo et al. [105] fabricated a monolithic FRT sensor using a TiO_2_ film sandwiched between a PET substrate and the molecular sieve ZIF-7/polyether block amide (PEBA) hybrid overlayer, as shown in Fig. 10a. The obtained pristine TiO_2_ sensor showed an ultrahigh response to 5 ppm formaldehyde (Sensitivity (S) = 5,041.0) and ethanol (S = 1,1264.3) under UV illumination (0.184 mW). In contrast, the sandwich-shaped sensor showed a large response to 5 ppm formaldehyde (S = 1350.9), while ethanol response (S = 23.5) became negligible, showing an exclusive detection of formaldehyde, as shown in Fig. 10b. This result demonstrated that the molecular-sieving effect of ZIF-7/PEBA, successfully sieving formaldehyde by ethanol filtration. The UV-irradiation not only provided a ppb-level formaldehyde detection capability with LOD of 3.8 ppb, but also laid a solid foundation for molecular sieve to improve the selectivity of formaldehyde sensor with an ultrahigh response. Furthermore, the performance of sensors is barely affected after bending 200 times or bending at different angles. The outstanding robustness could be contributed to the superior sandwich structure.

Apart from the UV illumination, visible-light illumination assisted FRT gas sensors with improved response have been presented. In particular, visible-light illumination has attracted numerous attentions as visible light is an environmental-friendly and easily accessible light source for portable gas sensors. Geng et al. [70] fabricated lamellar layered ZnO_1−x_ FRT NO_2_ gas sensors on polypropylene (PP) substrates through suspension flame spraying (SFS) method. The bandgap of ZnO was successfully narrowed down by SFS method due to the high heating/cooling rates and reducing atmosphere. As a result, the light absorption was successfully shifted from the UV to the visible light region. In addition, ascribed to high volume-to-surface ratio and the lamellar hierarchical porous nanostructure, the ZnO_1−x_ nanofilms showed a linear response to 0.25–1 ppm NO_2_ under white-light illumination, as shown in Fig. 10c. In addition, a flexible all-inorganic RT MOS gas sensor based on yttria-stabilized zirconia (YSZ)/In_2_O_3_/graphitic carbon nitride (g–C_3_N_4_) (ZIC) [66] was reported, employing a visible-light powered scheme to lower the operating temperature, using fibrous ceramic YSZ as the flexible substrate and In_2_O_3_/g–C_3_N_4_ as the activated sensing region. The promoting effect of visible light on sensing performance was further studied. The dynamic NO_2_ responses of YSZ/In_2_O_3_ (ZI) and ZIC flexible sensors at RT with or without visible-light illumination are shown in Fig. 10d, e. It can be observed that in the absence of light irradiation, the response is low and the recovery ability is negligible. In contrast, under visible light irradiation, the sensor exhibited sharply increased response after being exposed to NO_2_ and the response fully recovered to original value after removing NO_2_. Therefore, visible-light irradiation can not only effectively improve the gas sensing response, but also prompting response/recovery process.

In brief, photoactivation is effective in reducing the operating temperature and provides a distinctive way to realize FRT MOS gas sensors. The mechanism of photoactivation is based on the activation of photogenerated electron–hole pairs generated by the photoelectric effect. The specific preparation process can reduce the MOS material bandgap, which shifts the light absorption from the UV region to the visible region. Both UV and visible light irradiation can effectively reduce the operating temperature, increase the response value, and speed up the response/recovery process, which is another perspective to achieve RT operation in addition to material design.

## Applications of the FRT Gas Sensors Based on MOS

Gas sensors are currently used in a wide range of applications in smart homes, food safety monitoring, public safety, healthcare, medical diagnostics, environmental protection, and industrial/agricultural monitoring. Some emerging applications of gas sensing include ingestible electronic capsules, electronic skin, and smart electronic nose, imposing higher requirements on the low operating temperature, high gas sensing properties, excellent interference immunity, and stable mechanical flexibility of the gas sensors. Ingestible electronic capsules are non-invasive capsules that are delivered to the human gastrointestinal tract to detect various gases such as methane, oxygen, hydrogen, and carbon dioxide for human health detection [229, 230]. Gas sensors that exhibit high performance at RT are considered to be one of the key components of ingestible electronic capsules. In addition, the natural skin uses ion reporting signals, while the flexible, comfortable and stretchable electronic skin uses electron reporting signals. The electronic skin equipped with stretchable gas sensors can be perfectly fitted to the human body to achieve real-time detection of environmental gases. FRT gas sensors can effectively reduce the energy consumption, simplify the structure, and be applicable for harsh environments, such as for the detection of flammable explosives and various low-temperature operating wearable devices. The traditional MOS materials are hindered by the disadvantage of high operating temperature for wearable applications. The MOS-based FRT gas sensors with high performance presented in this paper are most attractive for wearable device applications. Currently, MOS-based FRT gas sensors have been reported for multi-detection sensor arrays and wearable devices such as smart masks, gas sensing watches, E-textiles, and skin-fitting RFID.

**Fig. 11:**
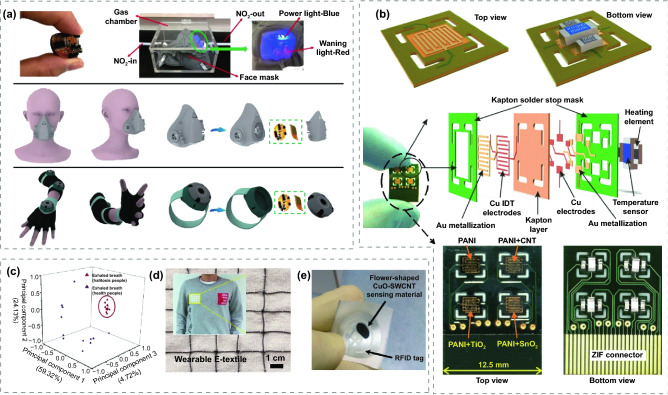
**a** The FRT NO_2_ monitoring system and wearable applications for smart masks and watches. Reproduced with permission from Ref. [231]. Copyright (2022) Springer Nature. **b** The expanded view, structural composition, and physical demonstration of the PANI-based sensor arrays. Reproduced with permission from Ref. [113]. Copyright (2018) IEEE. **c** The 3D feature space for principal component analysis of RGO/ZnO hybrid towards different exhaled gases from healthy individuals and patients with halitosis. Reproduced with permission from Ref. [98]. Copyright (2019) Elsevier. **d** The 5 × 5 gas sensors array networks of the E-textile. Reproduced with permission from Ref. [54]. Copyright (2019) American Chemical Society. **e** The optical image of fabricated wireless RFID sensor. Reproduced with permission from Ref. [112]. Copyright (2016) Elsevier

With urbanization and industrialization, air pollution levels are increasing. Monitoring of the public environment is necessary to protect people from the gaseous pollutants and toxic gases. In addition, it is significant to detect the concentration of toxic gases in industrial production in real time and to develop wearable gas detection and warning devices for workers, especially in environments where toxic gas leaks are likely to occur. Zhang et al. [231] deposited RGO/SnO_2_ sensing films on PI substrates by spraying process and integrated them to form a FRT NO_2_ monitoring system. The gas monitoring system can detect the lowest concentration limit of 20 ppm. When the blue indicator light turns on, the system is in operation. While the red warning indicator light turns up when the NO_2_ gas concentration exceeds the set threshold. The NO_2_ monitoring system can also be embedded in masks and watches owing to its small volume and excellent flexibility, realizing practical applications of flexible NO_2_ gas sensors in the field of wearable electronics (Fig. 11a). Gao et al. [100] prepared SWCNTs, MWCNTs, and ZnO quantum dot-modified SWCNTs as sensing materials on flexible nylon fibers, which could be further integrated into the mask by weaving. The smart mask selectively detects NH_3_, HCHO, and C_2_H_5_OH by sensing the change of sensor resistance to control the corresponding LEDs to turn on (Fig. 4f).

E-noses are devices used for sniffing out gaseous compounds in the environment like the human olfactory organ. E-noses typically consist of numerous chemical gas sensors, which are modulated by signal and pattern recognition techniques so that the sensor arrays can respond to a range of gases [232]. The selectivity is achieved by analyzing the characteristic patterns of response data in the sensor arrays. The integrated configuration of gas sensor arrays might be the future trend for constructing stable and efficient E-noses. Kroutil et al. [113] prepared pristine PANI, PANI/CNTs, PANI/SnO_2_ and PANI/TiO_2_ composites on PI flexible substrates. Four sensor elements were fabricated on a single flexible substrate, greatly facilitating a wider and convenient application. The expanded view, structural composition, and physical demonstration of their sensor arrays are shown in Fig. 11b. The RT gas sensing performance of the fabricated gas sensor arrays towards different gas atmospheres (O_2_, NH_3_, CO_2_, NO_2_, acetone, toluene, and relative humidity) was investigated. However, the sensor arrays did not further demonstrate effective discrimination of these gases, and the lack of selectivity is of concern and further investigation needs to be carried out. It is possible to combine with machine learning algorithms to achieve substantial enhancement, such as detect multiple gases and significantly improve the selectivity of the sensor arrays, which is unimaginable for the traditional sensors. A sensor array consisting of pristine SnO_2_, SnO_2_/PANI, RGO/PANI and SnO_2_/RGO/PANI was carried out by Zhang et al. [98] H_2_S with different concentrations (0.1–1.2 ppm) were apparently discriminated from other interfering gas species (NH_3_, ethanol, and acetone) processed by principal component analysis algorithm. Furthermore, the sensor arrays can effectively distinguish between the exhaled gas of a healthy people and the exhaled gas of halitosis patients with a detection limit of 0.1 ppm. The classification map is presented in Fig. 11c, where it can be seen that the exhaled breath patterns of healthy individuals are randomly dispersed, whereas the patterns of patients with halitosis are classifiable together, demonstrating its potential prospects for exhaled gas analyzers.

In smart wearable electronic devices, E-textiles are popular for their low price, light weight, and good compatibility with clothes. Li et al. [54] prepared RGO/ZnO nanosheets on cotton/elastic threads to form conductive sensing fibers, which are stretchable, twistable and bendable with excellent wearability and knittability. The sensing threads exhibited superior sensing performance to NO_2_ at RT with high response (44% to 50 ppm), low theoretical detection limit (43.5 ppb) and fabulous deformation tolerance. To further improve the reliability and expand the practical applications, the RGO/ZnO hybrid threads were woven into a 5 × 5 sensors array network (Fig. 11d). The response of the incorporated networks is close to the average response value of individual sensors, thus achieving an enhanced sensing reliability with a single measurement. More importantly, even if some of the individual sensors are out of operation after long-term use, the network can remain in working state, thus improving the repairability, long-term stability, and practicality of sensor array. The wearable e-textile operating at RT with great sensing performance, superior flexibility and scalable applications represent a novel perspective for extensive monitoring of human activities in human–machine interactions and mobile devices.

As the demand for low-cost, flexible, and low-energy wireless electronics and the growing interest in "cognitive intelligence", RFID is considered as one of the most revolutionary technologies to achieve ubiquitous IoTs. Asad et al. [112] drop-cast the prepared flower-like CuO-SWCNTs on the antenna of the commercial 13.56 MHz RFID tags (Fig. 11e). The obtained wireless RFID sensor was adhered with adhesive paste on the back side, which could be perfectly attached to the skin. What’s more, the RFID sensor can selectively detect H_2_S at RT with an ultralow LOD of 100 ppb and a long-term stability of 30 days, and exhibit excellent sensing performance under the bending situation. These results indicates that the RT-operating materials can be manufactured into flexible RFID sensors by extremely facile drop-casting process, demonstrating their possible wide application in healthcare and environmental monitoring.

## Conclusion and Future Perspectives

In this review, we have systematically analyzed and summarized the recent advances on FRT MOS gas sensors. Our attention concentrates on the critical review of FRT gas sensors, focusing on pristine MOS and the hybrids of MOS modified with nanostructure noble metal, organic polymers, carbon-based structures, and TMDCs materials. In addition, light-illuminated MOS-based FRT gas sensors are also summarized. Strategies for implementing FRT gas sensors are discussed from the viewpoints of material design, alternate activation, and mechanism explanation. The diverse types of sensors discussed above indicate that an excellent mechanically flexible gas sensor is the appropriate combination with flexible substrates (polymeric substrates, textiles, paper-based substrates, etc.), device structures and steady contact between substrates and sensing materials. At the meantime, the high-performance RT gas sensor is the justified combination with morphological nanostructures, hybrid materials, and synergetic interfacial effect. This review will have considerable reference value for design, fabrication and development of novel FRT MOS gas sensors.

Generally, MOS gas sensors have the merits of easy synthesis, high response value, low cost, rapid response/recovery, great reversibility, and excellent stability. However, their high-temperature operation hinders their wider applications. For example, when operating at RT, many MOS gas sensors exhibit inadequate sensitivity, reduced selectivity and the response/recovery process becomes slower, sometimes up to tens of minutes, which is critical for the rapid detection of hazardous gases and timely alarm triggering. The performance of MOS gas sensors can be enhanced by several methods, including preparation of various morphologies of MOS, modification of MOS with noble metal, composites with organic polymers, carbon-based materials and TMDCs materials, and light-illuminated assistance. The main conclusions are summarized as follows:

1. Nanostructures of various morphologies for pristine MOS have been prepared to help reducing the operating temperature to RT and enhancing their response value, response/recovery speed, selectivity, and LODs. Among them, 1D nanostructured sensing materials (nanofibers, nanowires, nanorods, etc.) are the preferred materials for RT gas sensors due to their inherent geometrically higher surface-to-volume ratio. Furthermore, 1D nanostructures not only contribute to the increased adsorption capacity to the analyte, but also provide more significant modulation of the electrical properties when exposed to the analyte attributed to a broader region of interaction in the cross-section.
2. Modification with noble metals is also a superior scheme to enhance the response and selectivity due to the synergistic effect of chemical sensitization and electronic sensitization. In addition, noble metal modification is generally effective in changing the surface nanostructure and morphology of MOS materials, increasing the number of active sites, creating more defects, generating more oxygen vacancies, providing more oxygen species, and enhancing surface lattice oxygen activity. Furthermore, Schottky barriers are formed when noble metals are in close contact with MOS materials, which can change the electron distribution and energy band structure in MOS materials. Moreover, some noble metals are specific for the detection of certain gases, which is advantageous for RT operation. Besides, the small size of noble metal nanoparticles does not affect the mechanical flexibility properties of the sensing material. All these processes can help to enhance the gas sensing performance of FRT MOS-based gas sensors.
3. Organic conducting polymers especially PANI has been widely used in MOS-based FRT gas sensors owing to their tunable electrical properties, excellent mechanical flexibility, RT operation, and morphological tunability. The protonation/deprotonation process of PANI and the formation of heterojunctions at the interface of PANI and MOS endow superior sensing properties. 0D nanoparticles, 1D nanofibers, and 2D nanosheets of PANI have been utilized for the preparation of high-performance FRT gas sensors. Among them, 0D PANI nanoparticles attached to MOS exhibit favorable catalytic enhancement. 1D PANI nanofibers show better RT sensing performance due to better conductivity and higher surface-to-volume ratio. PANI nanosheets with large surface area and tremendous mechanical stability showed no degradation after bending tests. Beyond that, the PANI-modified MOS sensing materials demonstrate fabulous demonstrate resistance due to the accelerated ES to EB transition process of PANI in high humidity environments. Generally, organic conducting polymers modified MOS FRT gas sensors exhibit high sensitivity and low LODs.
4. Various organic polymers such as chitosan, PVA, CMC, etc. are mixed with IL and MOS to form membranes, which can not only act as the flexible substrate, but also exhibit gas sensing characteristics. The presence of IL can effectively control the electrical conductivity of the organic–inorganic compounds and help improving the stretchability and flexibility of the membranes.
5. Carbon-based materials including 1D SWCNTs, MWCNTs, and 2D RGOs have been applied to MOS-based FRT gas sensors owing to high carrier mobility, unique flexibility properties, high surface-to-volume ratio, abundant adsorptive sites, and RT operation. The reported carbon-based materials modified MOS FRT gas sensors typically exhibit lower LODs, shorter response/recovery times, lower operating temperatures, and better mechanical flexibility. However, they suffer from the low significant increase in response value.
6. TMDCs-modified MOS FRT gas sensors take full advantage of the superior mechanically flexible lamellar nanostructure of TMDCs and the effective modulation of heterojunction between MOS and TMDCs, greatly improves the slow and low response of the original TMDCs. However, there are few papers proposed to fabricate the FRT gas sensor based on TMDC/MOS composite. The performance of TMDCs materials modified MOS FRT gas sensors remains to be concluded and discovered.
7. Light illumination is an alternate strategy to realize RT operation. The mechanism of light illumination is based on the activation of photo-generated electron–hole pairs generated by the photoelectric effect. When illumination, the surface of MOS generates photo-induced oxygen ions, which have weak bonding with MOS and can be easily reacted with target gas, resulting in an enhanced gas sensing performance. Both UV-light and visible-light illumination have been employed to manufacture FRT gas sensors, effectively reducing the operating temperature, increasing the response value, and speeding up the response/recovery process.

Although tremendous advances have been made in the development of FRT MOS gas sensors, many challenges and problems remain in achieving great mechanical flexibility, high response valve, rapid response/recovery, exclusive selectivity, low LODs, and long-term stability. The challenges and future perspectives involved in FRT gas sensors are summarized as follows:

- Various MOS have been applied to FRT gas sensors, including n-type MOS (SnO_2_, ZnO, TiO_2_, WO_3_, In_2_O_3_, Fe_2_O_3_, MoO_3_, CdO, CeO_2_, CoFe_2_O_4_ and SrGe_4_O_9_), and p-type MOS (CuO and Co_3_O_4_). However, there are still many sensitive and promising MOS that have not been applied to FRT gas sensors, such as V_2_O_5_, NiO, and MnO_2_, which have been extensively studied and behaved great gas sensing performance. The introduction of novel MOS might be helpful to find new well-behaved sensing materials to achieve FRT sensors.
- In general, most FRT MOS-based gas sensors are based on pristine MOS or two-component composites. Recently, ternary composites-based MOS gas sensors have been prepared to achieve superior performance. The substantial increase in sensing performance of ternary composites is mainly attributed to the formation of numerous easily modulated heterostructures at the interfaces of different components, the generation of more defects and active sites, and the increase in possible conducting channels. However, there are still few researches focusing on ternary or multivariate composites, which may have excellent prospects for FRT gas sensors.
- Flexible substrates have been developed relatively mature at present, exhibiting great mechanical property. Up to now, polymers (PI, PET, PDMS, nylon, etc.), textiles (cotton fabric, yarn, fiber, etc.), and paper-based substrates (cellulose, tattoo papers, etc.) have been widely used for flexible gas sensors. However, one of the key challenges is the cracking and spalling of the sensing component. Generally, flexible gas sensors are fabricated through simple spin-coating, dip-coating, and drying, which leads to the agglomeration, densification, and nonuniformity of the sensing component, deteriorating the long-term stability and mechanically flexibility of the flexible gas sensors. As new processing and fabricating techniques have emerged, the preparation of flexible gas sensors has also been innovated. Some preparation methods for in situ growth of sensing materials on flexible substrates reported in this review might increase the bonding between sensing materials and substrates, such as in situ hydrothermal process [92, 93], in situ sol–gel grown [104], in situ polymerization [79, 87], and in situ self-assembly method [83]. Several novel transfer methods have also been reported, such as suspension flame spraying [70, 76], hot pressing method [94], slide transfer, roll transfer, and heat transfer [90]. In addition, the sensing material and organic polymer solution are mixed and dispersed uniformly by stirring and ultrasonication, and then solution-cast technique is used to form a monolithic flexible sensing material [95–97, 103, 192], which is beneficial for the stability and longevity. What’s more, some specific additives such as sorbitol and glycerol might be helpful to enhance the extensibility and flexibility of the sensitive film. However, there is less discussion on how the preparation methods affects the flexible properties of the sensors, and the fracture mechanism of sensing films remains to be explored. According to different sensing materials, selecting suitable flexible substrates to reduce the elastic mismatch may be beneficial to fabricate flexible gas sensors with better mechanical properties.
- Up to now, TMDCs materials have been widely applied to RT gas sensors with low LODs, great response and rapid response/recovery. However, it is rarely combined with flexible substrates to form FRT sensors. TMDCs materials modified MOS might be promising materials for FRT gas sensors.
- Owing to its good electrical conductivity, carbon-based materials can expand electron pathways and accelerate electron transport when hybridized with MOS. At present, carbon-based materials modified MOS FRT gas sensors have excellent development prospects and have been reported in practical applications, such as smart face masks, smart textiles, and wireless RFID, which might be the earliest FRT gas sensors to be put into commercial products.
- At present, MOS-based FRT sensor array has been reported. It is worth believing that the integrated configuration of sensor array might be the future trend of the robust flexible gas sensing system. The sensor array can not only get a more accurate and reliable result through one-time detection, but also improve long-term stability of the system due to the configuration of multiple sensors. In addition, flexible gas sensor arrays in combination with machine learning algorithms to achieve multiple gases detection and significantly improve the selectivity of the sensor arrays, which is unimaginable for the traditional sensors. The integrated configuration of gas sensor arrays might be the future trend for constructing stable and efficient E-noses.
- Although light illumination has been demonstrated to be a useful method to enhance the performance of sensors, the mechanism behind it remains to be explored. Generally, ultraviolet light sources are expensive and might lead to photodegradation of sensing materials, limiting its extensive applications. Therefore, designing more sensitive materials that can be activated by visible light is more beneficial for practical applications.

To date, the development of FRT gas sensors has just started, and there are still many challenges and issues in achieving excellent flexibility and gas sensitive characteristics. In the future, as new materials, innovative processes and advanced structures are further developed, high-performance FRT gas sensors will be more facilely available.
